# USP14 modulates stem-like properties, tumorigenicity, and radiotherapy resistance in glioblastoma stem cells through stabilization of MST4-phosphorylated ALKBH5

**DOI:** 10.7150/thno.103629

**Published:** 2025-01-13

**Authors:** Xiao Zhou, Qiaoxi Xia, Botao Wang, Junjun Li, Bing Liu, Sisi Wang, Min Huang, Ronghui Zhong, Shi-Yuan Cheng, Xuan Wang, Xiaobing Jiang, Tianzhi Huang

**Affiliations:** 1 State Key Laboratory of Cellular Stress Biology, School of Life Sciences, Faculty of Medicine and Life Sciences, Shenzhen Research Institute of Xiamen University, Xiamen University, Xiamen, Fujian 361102, China.; 2 Department of Neurosurgery, Union Hospital, Tongji Medical College, Huazhong University of Science and Technology, Wuhan, Hubei 430022, China.; 3 The Ken and Ruth Devee Department of Neurology, Lou and Jean Malnati Brain Tumor Institute Northwestern Medicine, The Robert H. Lurie Comprehensive Cancer Center, Simpson Querrey Institute for Epigenetics, Northwestern University Feinberg School of Medicine, Chicago, IL60611, USA.

**Keywords:** Glioblastoma, USP14, ALKBH5, MST4, radioresistance

## Abstract

**Rationale**: Glioblastoma (GBM) is the most aggressive type of primary brain cancer and contains self-renewing GBM stem cells (GSCs) that contribute to tumor growth and therapeutic resistance. However, molecular determinants governing therapeutic resistance of GSCs are poorly understood.

**Methods**: We performed genome-wide analysis of deubiquitylating enzymes (DUBs) in patient-derived GSCs and used gene-specific shRNAs to identify an important DUB gene contributing to GSC survival and radioresistance. Subsequently, we employed mass spectrometry and immunoprecipitation to show the interaction between USP14 and ALKBH5, and identified the upstream kinase MST4, which is essential for the deubiquitylation and stabilization of ALKBH5. Additionally, we performed integrated transcriptome and m^6^A-seq analyses to uncover the key downstream pathways of ALKBH5 that influence GSC radioresistance.

**Results**: Our study demonstrates the essential role of the deubiquitinase USP14 in maintaining the stemness, tumorigenic potential, and radioresistance of GSCs. USP14 stabilizes the m^6^A demethylase ALKBH5 by preventing its K48-linked ubiquitination and degradation through HECW2. The phosphorylation of ALKBH5 at serine 64 and 69 by MST4 increases its interaction with USP14, promoting ALKBH5 deubiquitylation. Furthermore, ALKBH5 directly interacts with the USP14 transcript in a manner dependent on YTHDF2, establishing a positive feedback loop that sustains the overexpression of both proteins in GSCs. The MST4-USP14-ALKBH5 signaling pathway is crucial for enhancing stem cell-like traits, facilitating homologous recombination repair of DNA double-strand breaks, and promoting radioresistance and tumorigenicity in GSCs. This signaling cascade is further stimulated in GSCs following exposure to ionizing radiation (IR). Inhibiting USP14 with the small molecule IU1 disrupts ALKBH5 deubiquitylation and increases the effectiveness of IR therapy on GSC-derived brain tumor xenografts.

**Conclusion:** Our results identify the MST4-USP14-ALKBH5 signaling pathway as a promising therapeutic target for treating GBM.

## Introduction

Glioblastoma (GBM) is one of the deadliest cancers, with a median survival of under 15 months and a 5-year survival rate of only 5% 1. Studies on gene expression profiles have categorized IDH wild-type GBM into three subtypes: proneural (PN), classical (CL), and mesenchymal (MES) 2. The MES subtype is the most aggressive and is strongly associated to poor patient outcomes 2. Standard treatment for GBM patients involves careful surgical removal of the tumor, followed by radiation therapy and chemotherapy with temozolomide (TMZ). While these approaches can improve overall survival and maintain a good quality of life, tumor recurrence is still a frequent and often inevitable issue 3. A key feature of GBM tumor biology is the significant heterogeneity both within and between tumors, which includes GBM stem cells (GSCs) at the top of the tumor hierarchy. These cancer stem cells (CSCs) have a unique epigenetic profile that maintains their stem-like properties and contributes to resistance to treatment 4. GSCs exhibit altered metabolism and reprogrammed epigenomes, with their interactions driving GBM tumor initiation and progression 5-7. Thus, uncovering the molecular mechanisms that govern GSCs is crucial for developing new targeted therapies for GBM.

While significant knowledge exists about how DNA methylation affects transcription, new evidence indicates that epigenetic regulation is more complex than previously believed, with RNA also being subject to methylation. One prevalent modification, N^6^-methyladenosine (m^6^A), influences RNA behavior, and its abnormal changes are closely associated to the development and progression of human cancers 8, 9. m^6^A modification is a dynamic and reversible process regulated by methyltransferases (the "writers"), demethylases (the "erasers"), and m^6^A-binding proteins (the "readers") 10-12. m^6^A demethylases, such as FTO and ALKBH5, eliminate the m^6^A modification from RNA, thus regulating the overall levels of this modification 13. ALKBH5 is implicated in various cancers, including GBM, where its elevated expression in CSCs, promoting their self-renewal 14-16. This suggests that ALKBH5 could serve as a valuable biomarker and a potential target for CSC suppression. Although several inhibitors like IOX3 17, MV1035 18, ALK-04 19 have been developed to target ALKBH5, none have received clinical approval for cancer treatment, likely due to issues with target specificity, therapeutic effectiveness, safety, and pharmacokinetics. Therefore, a better understanding of the molecular mechanisms regulating ALKBH5 is essential for developing alternative strategies to target it in CSCs.

Ubiquitination is a widespread post-translational modification that is essential for regulating numerous critical cellular functions 20. Abnormal ubiquitination is increasingly linked to various diseases, including cancers 21. These ubiquitin modifications can be reversed by deubiquitylating enzymes known as deubiquitinases (DUBs). USP14, a member of the ubiquitin-specific proteases (USPs) family, is the sole DUB that interacts with the 19S proteasome to prevent substrate degradation by removing ubiquitin chains 22, 23. Recent studies highlight USP14's significant roles in various cancers and its potential as a cancer therapy target 24, 25. For instance, USP14 is necessary to inhibit TRIM21-mediated K48 ubiquitination of IDO1, leading to increased IDO1 levels in colorectal cancer and promoting tumor cell immune evasion 26. Additionally, USP14 deubiquitinates TAZ, enhancing its stability, and is positively regulated by TAZ-TEAD1/4, creating a feedback loop that drives tumor progression and metastasis in pancreatic ductal adenocarcinoma (PDAC) models 27. However, the specific functions of USP14 in GBM remain largely unexplored.

This study demonstrates that USP14 plays a role in the RNA m^6^A pathway and brain tumor biology by stabilizing ALKBH5. Mammalian sterile-20-like kinase 4 (MST4), also known as serine/threonine kinase 26, is activated by various cellular stresses and is crucial for cell survival and growth 28-30. The phosphorylation of ALKBH5 by MST4 is vital for its deubiquitylation and stabilization by USP14. We demonstrate that the MST4-USP14-ALKBH5 signaling pathway significantly influences the stem-like characteristics, homologous recombination (HR)-mediated repair of DNA double-strand breaks (DSBR), tumorigenicity, and radiation sensitivity of GSCs. Furthermore, the inhibition of this signaling pathway enhances the cytotoxic efficacy of radiotherapy in preclinical models of GBM.

## Results

### The expression of USP14 is enhanced in GSCs and exhibits a negative correlation with the prognosis of GBM patients

**Figure 1:**
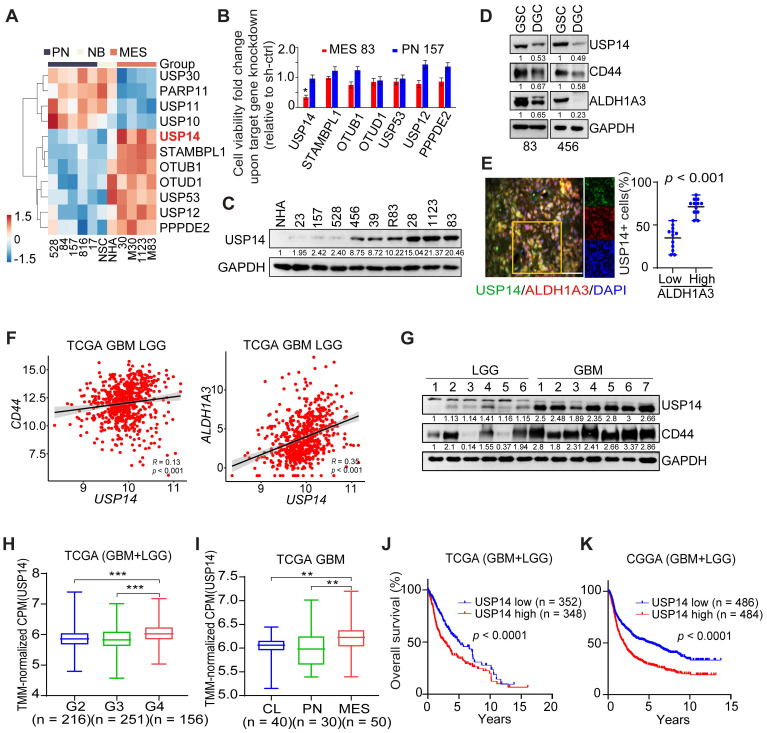
** Elevated levels of USP14 expression are associated with the tumorigenicity of patient-derived GSC cells.** (**A**) Heatmaps depicting the most differentially expressed genes encoding deubiquitinating enzymes in patient-derived PN and MES GSCs, neural progenitors (NSC), and normal astrocytes (NHA) (GEO: GSE67089). (**B**) Mean fold alteration in cellular viability after knockdown of indicated gene expression in GSC MES 83 and PN 157. (**C**) Immunoblotting (IB) analysis of USP14 protein expression levels in NHA and GSCs. (**D**) IB analysis for USP14, CD44, and ALDH1A3 in GSC 83 and 456 cells as well as their corresponding differentiated-GSC (DGC) cells. (**E**) Immunofluorescence (IF) analysis of USP14 (green), ALDH1A3 (red), and DAPI (blue for nuclei). Left panel: representative images from GBM samples (n = 5). Right panel: quantification of USP14-positive cells among ALDH1A3-positive versus ALDH1A3-negative populations. Scale bar = 50 μm. Error bars represent ± SEM. (**F**) Pearson correlation analysis of the relationship between USP14 expression levels and those of CD44 (left) and ALDH1A3 (right) within the TCGA GBM+LGG cohort. (**G**) IB analysis of USP14 protein expression levels in LGG and GBM tissues. (**H**) Analysis of USP14 expression levels across glioma tissues of grades 2, 3, and 4 within the TCGA dataset. (**I**) Evaluation of USP14 expression levels across GBM mesenchymal (MES), proneural (PN), and classical (CL) subtypes. (**J** and **K**) Kaplan-Meier survival assessments regarding USP14 expression in GBM and LGG within the TCGA (J) and CGGA (K) cohorts. Statistical analyses were determined by two-tailed Student's t-test (B, E), Pearson Correlation test (F), one-way ANOVA with Tukey's multiple comparisons test (H, I) and log-rank test (J, K). *p < 0.05, **p < 0.01, and ***p < 0.001*.* Data are representative of two independent experiments with similar results.

Our previous study characterized a set of GSCs to investigate dysregulated signaling pathways. MES-like GSCs exhibit more aggressive phenotypic characteristics in comparison to PN-like GSCs 31. Based on the transcriptome array analyses (GEO: GSE67089; http://www.ncbi.nlm.nih.gov/geo), we compared the genome-wide expression levels of 122 deubiquitylating enzymes (DUBs), and found that seven DUB genes (USP14, STAMBPL1, OTUB1, OTUD1, USP53, USP12 and PPPDE2) were significantly upregulated in MES-like GSCs, relative to PN-like GSCs, normal neural stem cells and normal astrocytes (NHA) (Figure 1A). Upon knockdown (KD) with sequence-specific short hairpin RNA (shRNA), USP14 was also the only DUB that was required for cell survival of MES GSCs, rather than PN GSCs as judged by the decreased cell viability (Figure 1B). We found that USP14 was undetectable in NHA, and weakly expressed in PN-like GSC 23, 157, 528 cells, but was expressed at high levels in MES-like GSC28, 1123, 83 and unclassified GSC 456, 39, R83 cells (Figure 1C). Like two known MES GSC markers CD44 and ALDH1A3, expression level of USP14 was markedly decreased in differentiated GSCs (DGC) when compared to their GSC counterparts (Figure 1D). Additionally, USP14 was preferentially expressed in cells positive for ALDH1A3 in GBM tumor samples (Figure 1E). In TCGA glioma samples, *USP14* showed positive correlation with *CD44* and* ALDH1A3*, and inverse correlation with PN markers, *SOX2* and *OLIG2* (Figure 1F and S1A-B). Consistently, USP14 protein was highly expressed in GBM, compared with low-grade gliomas (LGG) tissues (Figure 1G). Analysis of *USP14* expression in the TCGA or CGGA glioma dataset revealed that increased expression of *USP14* correlated with glioma tumor progression (Figure 1H), with MES compared with CL and PN GBM subtypes (Figure 1I), and with decreased patient survival (Figure 1J-K). Furthermore, multivariate analyses revealed an inverse survival association with *USP14* expression even when accounting for *TP53* and *IDH1* mutation status, patient age as well as gender (Figure S1C-F). Additional examination of methylated status within the *USP14* promoter revealed no appreciable difference between PN and MES GSCs (Figure S1G).

### USP14 regulates cell growth, self-renewal, radioresistance, and tumorigenicity of GSCs

**Figure 2:**
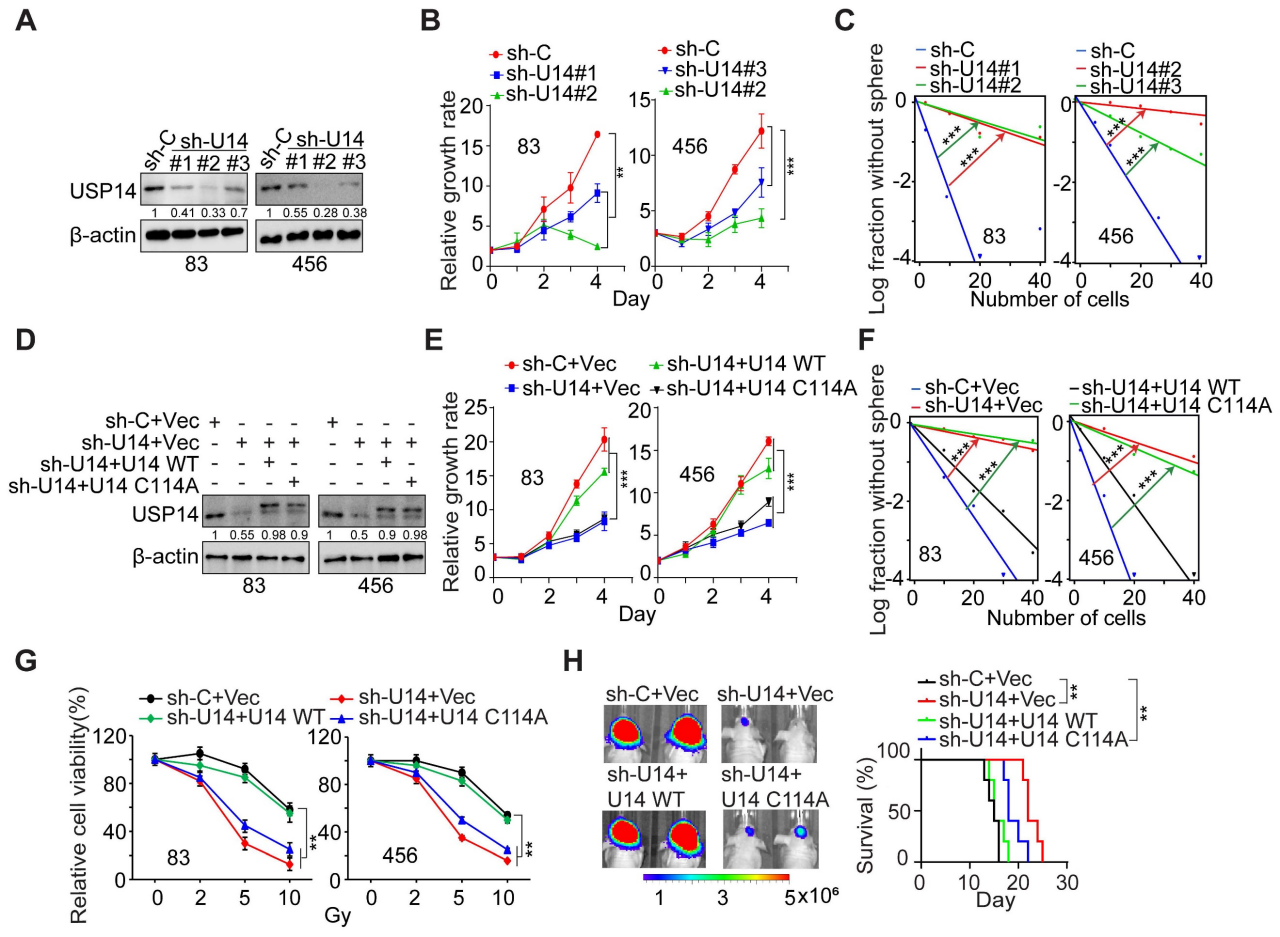
** USP14 regulates the proliferation, glioma sphere formation, radioresistance, and tumorigenicity of GSCs.** (**A**) IB analysis of USP14 protein levels in GSC 83 and 456 cells with indicated modifications. (**B** and **C**) Impact of USP14 silencing on the cell growth (B) and sphere-forming frequency (C) of GSC 83 and GSC 456 cells. (**D**) IB of USP14 in GSC 83 and 456 cells expressing sh-C or sh-USP14 (targeting 3´UTR of *USP14* mRNA), with or without re-expression of an shRNA-resistant USP14-WT (wild-type) or an inactive USP14 mutant C114A. (**E** and **F**) Cell proliferation (E) and sphere-forming frequency (F) of GSC 83 and 456 cells with indicated modifications. (**G**) Cell viability for GSC 83 and 456 cells with indicated modifications after IR treatments. (**H**) *In vivo* Bioluminescence imaging (BLI) GBM xenografts derived from the luciferase-labeled GSC 83 with indicated modifications (left panel). Kaplan-Meier survival curves of mice intracranially injected with the indicated GSC 83 cells (n = 5; right panel). The colored scale bars in panel (H) denote photon flux density in photons/s/cm²/steradian. Statistical significance was determined by two-way ANOVA test (B, E and G), the likelihood ratio test (C, F) and the log-rank test (H). All bar plot data are means ± SEM. **p < 0.01, ***p < 0.001. Data are representative of two to three independent experiments with similar results.

We used three distinct shRNA constructs to downregulate endogenous USP14 expression in GSC 83 and 456 cell lines (Figure 2A). The KD of USP14 markedly impeded the proliferation and sphere-forming capacity of GSC cells *in vitro* (Figure 2B-C and S2A-B). Following this, we reintroduced wild-type USP14 and a catalytically inactive USP14 mutant (USP14 C114A) into the two GSC lines with endogenous USP14 KD (Figure 2D). The restoration of USP14-WT, in contrast to USP14 C114A, rescued GSC cell proliferation, sphere-forming frequency, and radioresistance (Figure 2E-G), as well as reacquired the ability for intracranial tumor xenograft growth, ultimately diminishing the overall survival of tumor-bearing hosts (Figure 2H). These results suggest that USP14 is required for the growth, maintenance of stemness, radioresistance, and tumorigenic potential of GSCs.

### USP14 interacts with and deubiquitinates ALKBH5

**Figure 3:**
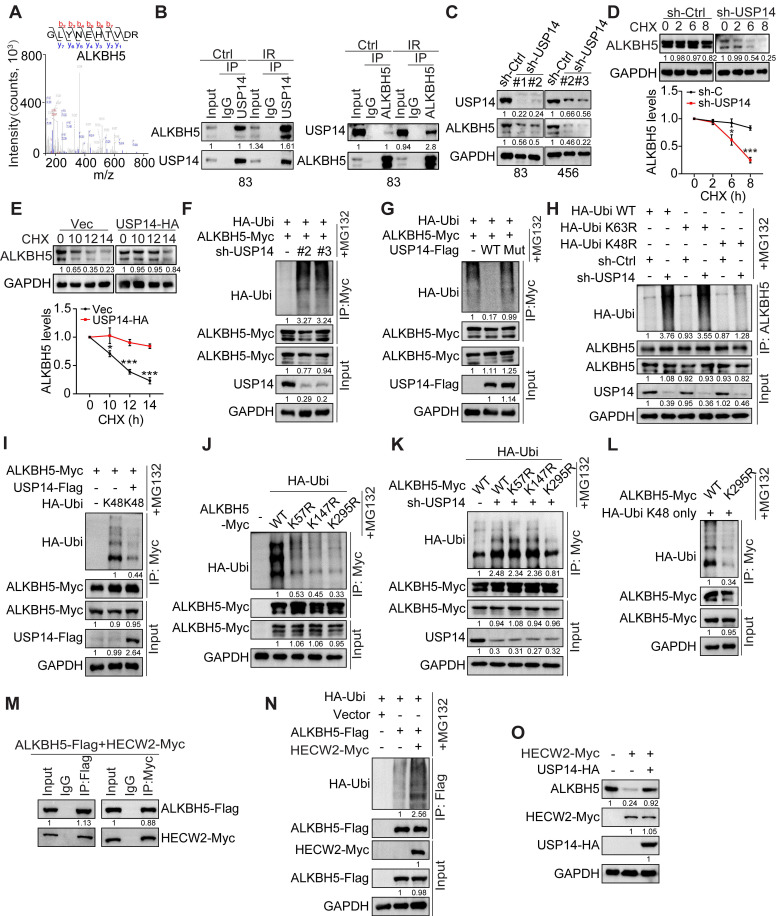
** USP14 directly binds and deubiquitylates ALKBH5.** (**A**) Identification of ALKBH5 in the USP14 -interacting proteins immunoprecipitated from HEK293T cells by mass spectrometry analysis. (**B**) Immunoprecipitation (IP)-IB analyses for USP14-ALKBH5 interaction in GSC 83 cells with or without IR treatment (2 Gy). IgG: immunoglobulin G. (**C**) IB analysis of USP14 and ALKBH5 in GSC 83 and 456 cells with indicated modifications. (**D** and** E**) IB analysis of ALKBH5 protein levels in GSC83 cells with or without USP14 knockdown (D) and in GSC23 cells with or without USP14 overexpression (E). Cells were treated with 50 μg/mL cycloheximide (CHX) for indicated time periods. The relative intensities of ALKBH5 protein bands were quantified against untreated controls. (**F-L**) ALKBH5 ubiquitination assay in HEK293T cells expressing indicated plasmids. (**M**) IP-IB analyses for ALKBH5-HECW2 interaction in HEK293T cells. (**N**) ALKBH5 ubiquitination assay in HEK293T cells with indicated modifications. (**O**) IB analysis of ALKBH5 in HEK293T cells with overexpression of HECW2 and/or USP14. In (F-L, N), cells were incubated with MG132 for 6 h before collecting for ubiquitination assays. Statistical significance was determined by two-tailed Student's t-test (D, E). Data are representative of two to three independent experiments with similar results. All data presented in the bar graphs are expressed as means ± SEM. *p < 0.05, **p < 0.01.

To elucidate the molecular mechanisms underlying USP14 in GBM, we performed mass spectrometry analysis on proteins that co-precipitated with Flag-tagged USP14 in HEK293T cells. Among the prominent candidates identified, the m^6^A demethylase ALKBH5 was detected in USP14 co-immunoprecipitation. Their interaction was confirmed and significantly increased in response to IR-induced DNA damage in GSC 83 and 456, as indicated by the expression of phospho-Histone 2A.X (γH2AX), a DNA damage marker, and the appearance of γH2AX foci 2 h after IR treatment (Figure 3A-B and S3A-B).

Depletion of USP14 resulted in a pronounced reduction in ALKBH5 protein levels and diminished the half-life of ALKBH5 upon treatment with cycloheximide (CHX) (Figure 3C-D), while it did not alter the mRNA levels of *ALKBH5* (Figure S3C). Conversely, overexpression of USP14 significantly enhanced the stability of ALKBH5 proteins (Figure 3E). KD of USP14 led to an increase in the ubiquitination of ALKBH5 (Figure 3F), whereas the overexpression of USP14-WT, but not USP14-C114A, effectively mitigated ALKBH5 ubiquitination (Figure 3G). Furthermore, co-localization studies demonstrated the interaction of USP14 and ALKBH5 within GSCs (Figure S3D). Interaction domain mapping indicated that the C-terminal domain of USP14 (amino acids 351-494) and the N-terminal sequences of ALKBH5 (amino acids 1-66) are critical for their association (Figure S3E-H).

Subsequently, we investigated the role of USP14 in modulating ALKBH5 through mechanisms dependent on K48 or K63 ubiquitination. USP14 KD led to a pronounced increase in K63R-linked polyubiquitination of ALKBH5, while K48R-linked polyubiquitination remained unaffected (Figure 3H). In contrast, the overexpression of USP14 resulted in a significant reduction of K48 ubiquitination of ALKBH5 (Figure 3I). Previous studies have indicated that ALKBH5 undergoes ubiquitination at residues K57, K147, and K295 32, and ALKBH5 K57R, K147R, and K295R mutants displayed diminished ubiquitination levels compared to wild-type ALKBH5 (Figure 3J). Notably, the ubiquitination status of ALKBH5 K295R was also influenced by USP14 KD when compared to wild-type and the K57R and K147R mutants (Figure 3K), indicating that K295 serves as a critical ubiquitination site affected by USP14. Additionally, the K295R mutation significantly reduced K48-linked polyubiquitination (Figure 3L) and conferred increased stability to ALKBH5 relative to wild-type ALKBH5 (Figure S3I). E3 ubiquitin ligase 2 (HECW2) was identified as a potential interacting partner of ALKBH5 33. We validated the interaction between ALKBH5 and HECW2 via co-immunoprecipitation (co-IP) (Figure 3M) and observed that HECW2 overexpression markedly augmented the ubiquitination of ALKBH5 (Figure 3N). In HEK293T cells, overexpression of HECW2 resulted in a decrease in ALKBH5 protein levels (Figure 3O), whereas co-overexpression of USP14 mitigated the reduction in ALKBH5 levels induced by HECW2 (Figure 3O). Collectively, these findings suggest that USP14 plays a crucial role in safeguarding ALKBH5 from ubiquitination and degradation mediated by HECW2.

### USP14 is required for IR-induced upregulation of ALKBH5

**Figure 4:**
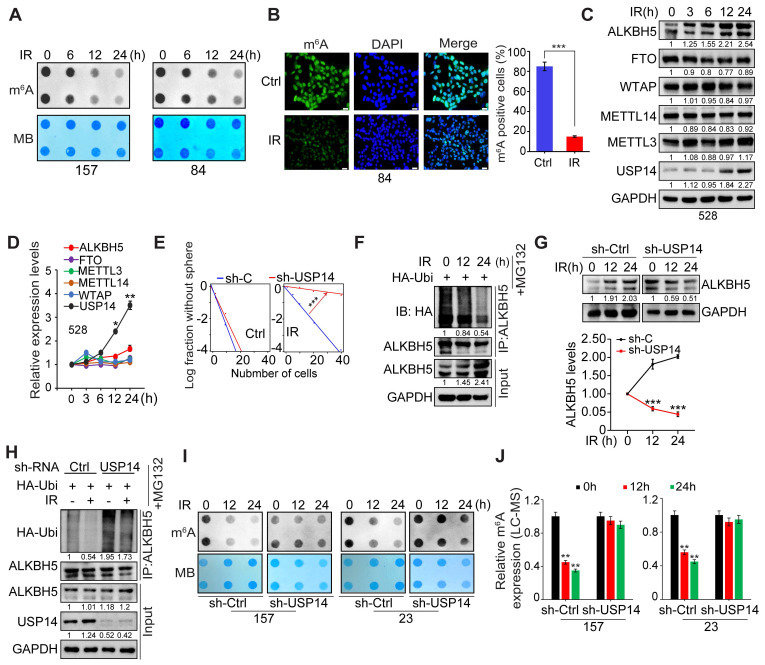
** USP14 is essential for the stabilization of ALKBH5 induced by IR, leading to a reduction in m^6^A levels in GSCs.** (**A**) Level of m^6^A were assessed via RNA dot blot in GSC157 and 84 cells with IR treatment for the indicated time. Methylene blue detected RNA loading. (**B**) IF for m^6^A in GSC84 cells with or without IR treatment. Scale bar = 50 μm. (**C**) IB analysis of ALKBH5, FTO, METTL3, METTL14, WTAP, and USP14 in GSC528 cells treated with IR for the indicated time. (**D**) quantitative real-time PCR (qPCR) for mRNA levels of *ALKBH5*, *FTO*, *METTL3*, *METTL14*, *WTAP*, and *USP14* in GSC528 cells treated with IR for the indicated time. Data were normalized to that in untreated cells, with GAPDH as an internal control. (**E**) Sphere-forming frequency for GSC 528 cells at day 5 after IR treatment. (**F**) ALKBH5 ubiquitination assay in GSC528 cells expressing HA-tagged ubiquitin. Cells were collected at indicated time points after IR treatment. (**G**) IB analysis of ALKBH5 protein levels in GSC 23 cells with and without the knockdown of USP14. Cells were harvested at indicated time after IR treatment, and the intensity of the ALKBH5 protein bands was quantified in comparison to the untreated control group. (**H**) ALKBH5 ubiquitination assay in GSC23 cells with indicated modifications and treatments. (**I** and **J**) levels of m^6^A levels were assessed by dot blot (I) and LC-MS (J) in GSC157 and 23 cells with indicated modifications and time after the IR treatment. In (A-J), IR treatment, 4 Gy. Statistical significance was determined by two-tailed Student's t-test (B, G), one-way ANOVA with Tukey's multiple comparisons test (D, J) and the likelihood ratio test (E). *p < 0.05, **p < 0.01, and ***p < 0.001. Data are representative of two to three independent experiments with similar results, with all bar plots shown as means ± SEM.

Considering the enhanced interaction between ALKBH5 and USP14 following ionizing radiation (IR) treatment, we aimed to evaluate the effects of IR on the stability of ALKBH5 and m^6^A modifications in GSCs. Through m^6^A dot blot assays and immunofluorescence (IF) staining, we observed a significant reduction in global mRNA m^6^A methylation in GSC157 and GSC84 cell lines post-IR exposure (Figure 4A-B). It is well-established that m^6^A modification is regulated by “writers” (METTL3, METTL14, WTAP) and “erasers” (ALKBH5, FTO). Notably, IR treatment led to a pronounced increase in the protein levels of ALKBH5 and USP14 in GSCs (Figure 4C), while the protein levels of METTL3, METTL14, WTAP, and FTO remained unchanged (Figure 4C). Real-time PCR analysis indicated that IR resulted in an approximate 3.5-fold elevation in *USP14* mRNA levels, with negligible effects on the mRNA levels of *ALKBH5* and other m^6^A regulatory genes (Figure 4D). To explore the function of USP14 in GSCs' response to IR, we performed KD of USP14 induced by IR. Our findings revealed that silencing USP14 augmented the anti-tumoral efficacy of IR, as evidenced by a decrease in GSC sphere formation frequency (Figure 4E). This suggests that USP14 plays a pivotal role in mediating GSC resistance to IR treatment.

Consistent with the elevated levels of ALKBH5 protein, we noted that IR significantly diminished the ubiquitylation of ALKBH5 in the presence of the proteasome inhibitor MG132. Conversely, the KD of USP14 hindered the deubiquitylation and stabilization of ALKBH5 prompted by IR (Figure 4F-H), as well as the demethylation of m^6^A (Figure 4I-J). Exposure to IR also increased the interaction between ALKBH5 and USP14, accompanied by an increase in the phosphorylation of serine/threonine residues within ALKBH5, rather than in USP14 (Figure S4A). Furthermore, treatment with λ-protein phosphatase on GSCs attenuated the IR-induced phosphorylation of Ser/Thr in ALKBH5, which subsequently disrupted the interaction with USP14. It is well-recognized that ATM, ATR, and DNA-PK play critical roles in the DNA damage response elicited by IR 34. However, the inhibition of these kinases using specific inhibitors did not alter the association between USP14 and ALKBH5 in GSCs (Figure S4B). These observations indicate that the IR-induced interaction between ALKBH5 and USP14 may be modulated by the phosphorylation status of the ALKBH5 protein.

### MST4 phosphorylates ALKBH5 and enhances its deubiquitylation and stabilization via USP14

**Figure 5:**
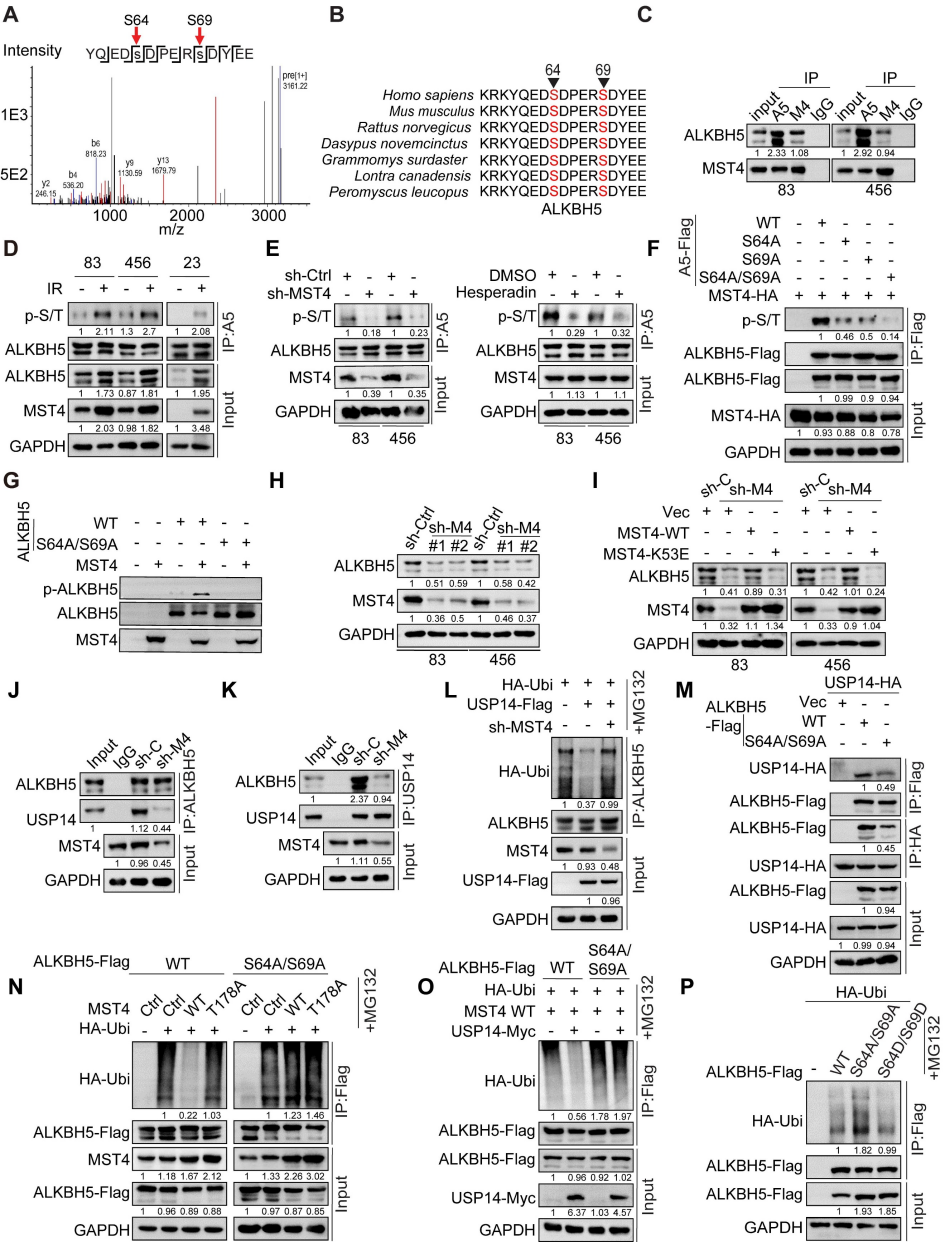
** MST4-dependent phosphorylation of ALKBH5 promotes the binding and deubiquitylation of ALKBH5 by USP14.** (**A**) Identification of phosphorylated S64/S69 peptides in human ALKBH5 protein from GSC 83 cells by mass spectrometry analysis. (**B**) Amino acid sequences surrounding S64/S69 in ALKBH5 across different species. Triangles at the top, serine residue that is conserved across species. (**C**) IP-IB analysis for the interaction between ALKBH5 and MST4 in GSC 83 and 456 cells. (**D**) IP-IB analysis of ALKBH5 phosphorylation, MST4 and ALKBH5 protein expression levels, in GSC 83, 456 and 23 cells with or without IR treatment. (**E**) IP-IB analysis of ALKBH5 phosphorylation in GSC 83 and 456 cells expressing sh-Ctrl or sh-MST4 (left), with or without treatment of hesperadin (right). (**F**) IP-IB assays of ALKBH5 phosphorylation in HEK293T cells expressing indicated plasmids. (**G**) *In vitro* kinase assays of MST4-WT, with ALKBH5-WT or the ALKBH5-S64A/S69A mutant as substrates. The resulting reaction mixtures were analyzed via IB using antibodies against p-ALKBH5, ALKBH5, and MST4. (**H** and** I**) IB analysis of ALKBH5 and MST4 in GSC 83 and 456 cells with indicated modifications. (**J** and** K**) IP-IB analysis for the interaction between endogenous USP14 and ALKBH5 in GSC 83 cells expressing sh-Ctrl or sh-MST4. (**L, N-P**) ALKBH5 ubiquitination assay in HEK293T cells with indicated modifications. (**M**) IP-IB for ALKBH5-USP14 interaction in HEK293T cells with the indicated modifications. Data represents three independent experiments with similar results.

Subsequently, we conducted mass spectrometry analysis on proteins immunoprecipitated with an antibody specific to ALKBH5 in GSCs. We detected phosphorylation of ALKBH5 at serine residues 64 and 69 (S64/S69), and observed that the amino acid sequence surrounding S64/S69 is highly conserved across various species (Figure 5A-B). Among the proteins that co-precipitated with ALKBH5, MST4/STK26 emerged as the most significantly enriched kinase. We validated the physical interaction between endogenous ALKBH5 and MST4 proteins in GSC83 and 456 cell lines, corroborating our mass spectrometry findings (Figure 5C). Furthermore, we found that ALKBH5 co-localized with MST4 within GSCs (Figure S5A).

Consistent with an earlier finding 30, this study revealed that IR significantly upregulated the expression of MST4, which correlated with an increase in phosphorylation of ALKBH5 (Figure 5D). To investigate whether MST4 directly phosphorylates ALKBH5, we employed MST4 KD or inhibited MST4 activity using the specific inhibitor Hesperadin in GSCs 35. Our results indicated that inhibition of MST4 notably diminished ALKBH5 phosphorylation levels (Figure 5E). Furthermore, overexpression of the wild-type MST4 (MST4 WT), in contrast to a catalytically inactive MST4 mutant (K53E), facilitated the phosphorylation of ALKBH5 (Figure S5B). To substantiate the phosphorylation at residues S64 and S69 of ALKBH5 by MST4, we co-expressed MST4 alongside FLAG-tagged wild-type ALKBH5 or non-phosphorylatable mutants S64A, S69A, and the double mutant S64A/S69A in HEK293T cells. The wild-type ALKBH5, as well as the S64A and S69A mutants, were phosphorylated by MST4, whereas the S64A/S69A double mutant showed no phosphorylation by MST4 (Figure 5F). Similar results were observed in ALKBH5 deficient GSC83 cells reconstituted with ALKBH5-WT, S64A, S69A or S64A/S69A (Figure S5C). To confirm the phosphorylation of ALKBH5 at the S64 and S69 residues, we developed a specific anti-p-ALKBH5 antibody that selectively recognized the phosphorylated S64/S69-ALKBH5 peptide and did not bind to a control non-phosphorylatable peptide (Figure S5D). Additionally, the overexpression of exogenous ALKBH5 in HEK293T cells resulted in a substantial increase in p-ALKBH5 levels, while the KD of endogenous ALKBH5 in GSC83 cells led to a significant reduction in p-ALKBH5 protein levels (Figure S5E). In HEK293T cells, overexpression of MST4-WT, as opposed to the kinase-dead mutant K53E, enhanced the phosphorylation of ALKBH5 (Figure S5F). The MST4-mediated phosphorylation at S64/S69 of ALKBH5 was further confirmed by *in vitro* kinase assays utilizing purified recombinant proteins of MST4-WT, ALKBH5-WT, and the non-phosphorylatable ALKBH5-S64A/S69A mutant (Figure 5G).

### MST4 stabilizes ALKBH5 protein by phosphorylating ALKBH5

Given the observed upregulation of MST4 and phosphorylated ALKBH5 (p-ALKBH5) after IR treatment, alongside the elevated endogenous ALKBH5 protein levels (Figure 5D), we proceeded to investigate the potential influence of MST4 and p-ALKBH5 on ALKBH5 protein stability. KD of MST4 markedly reduced the expression levels of ALKBH5 protein, whereas the overexpression or reconstitution of MST4-WT, but not the K53E mutant, preserved ALKBH5 protein levels. (Figure 5H-I and S5G). MST4 KD resulted in a reduction of endogenous ALKBH5 levels due to the ubiquitination-mediated degradation of ALKBH5; however, this suppressive effect of MST4 KD on ALKBH5 was counteracted by the application of the proteasome inhibitor MG132 (Figure S5H-I). MST4 KD decreased, whereas MST4 overexpression enhanced ALKBH5 stability (Figure S5J-K). Furthermore, MST4 KD in GSC83 cells attenuated the interaction between endogenous USP14 and ALKBH5, and suppressed the deubiquitylation of ALKBH5 by USP14 (Figure 5J-L). Compared with MST4-WT, MST4-K53E significantly decreased the interaction between USP14 and ALKBH5 (Figure S5L). Compared to the ALKBH5 WT, the non-phosphorylatable ALKBH5 S64A/S69A variant exhibited a reduced affinity for USP14 and a diminished deubiquitylation by MST4 and USP14, thereby resulting in decreased stability of the ALKBH5 protein (Figure 5M-P and S5M). Conversely, the phosphorylation-mimetic ALKBH5 S64D/S69D mutant significantly attenuated ALKBH5 ubiquitylation and preserved its stability (Figure 5P and S5M). Finally, we evaluated the impact of MST4 KD on IR-induced deubiquitylation of ALKBH5 mediated by USP14. MST4 KD attenuated the IR-enhanced ALKBH5-USP14 interaction and compromised ALKBH5 stability, while it augmented the ALKBH5-HECW2 interaction and increased ALKBH5 ubiquitination (Figure S5N-P). These findings indicate that MST4-mediated phosphorylation of ALKBH5 is crucial for its deubiquitylation by USP14.

### MST4 and USP14 regulate the expression of ALKBH5 target genes and self-renewal of GSCs through ALKBH5

To gain a comprehensive understanding of the regulatory function of ALKBH5 in GSCs, we performed an integrative analysis using m^6^A-seq (GSE93054) and RNA-seq (GSE87515) datasets 14. We analyzed the gene expression profiles of GSCs following the KD of ALKBH5 and identified that transcripts exhibiting changes in m^6^A methylation and expression levels after ALKBH5 depletion are predominantly associated with protein metabolism, double-strand break repair (DSBR) pathways, and the cell cycle (Figure S6A-C).

Given the association of ALKBH5 and energy metabolism pathways such as the mTOR pathway 36-38, we employed the CRISPR/Cas9 gene editing system to create ALKBH5-knockout (KO) GSC 83 cell lines, and explore the function of ALKBH5 in cellular metabolism through glycolysis/OXPHOS analyses with Seahorse extracellular measurements. The results revealed that ALKBH5-deficient GSCs exhibited a modest reduction in the extracellular acidification rate (ECAR), indicative of overall glycolytic activity, relative to wild-type GSCs. However, the absence of ALKBH5 did not significantly affect the oxygen consumption rate (OCR), a measure of mitochondrial oxidative phosphorylation (Figure S6D-E).

Translation serves as a crucial mechanism in protein metabolism, modulating the global expression levels of gene products 39. To assess the influence of ALKBH5 on translation, we conducted polysome profiling alongside RNA sequencing in both control and ALKBH5-KO GSC 83 cells. The results indicated that ALKBH5 KO significantly modified the distribution of polysomes, as evidenced by an elevation in the levels of 40S and 80S ribosomal subunits, alongside a corresponding reduction in 60S subunits, while the polysome fraction remained largely unchanged (Figure S6F), suggesting that the loss of ALKBH5 impaired translational efficiency in GSCs. We further evaluated the polysome sequencing data by normalizing polysome-associated RNAs against total RNA, enabling the systematic identification of transcripts exhibiting differential translation efficiency. Ultimately, we identified 6706 transcripts with enhanced translational efficiency due to ALKBH5 KO, while 2574 transcripts were downregulated. Pathway analysis revealed that transcripts actively translated into proteins were enriched in apoptosis and negative regulation of cell proliferation. In contrast, transcripts exhibiting buffering were enriched in biological pathways linked to homologous recombination, double-strand break repair, positive regulation of canonical Wnt signaling pathway and stem cell maintenance (Figure S6G). These findings imply that ALKBH5 facilitates the selective translation of mRNAs encoding pathways related to stemness and resistance to therapeutic interventions.

**Figure 6:**
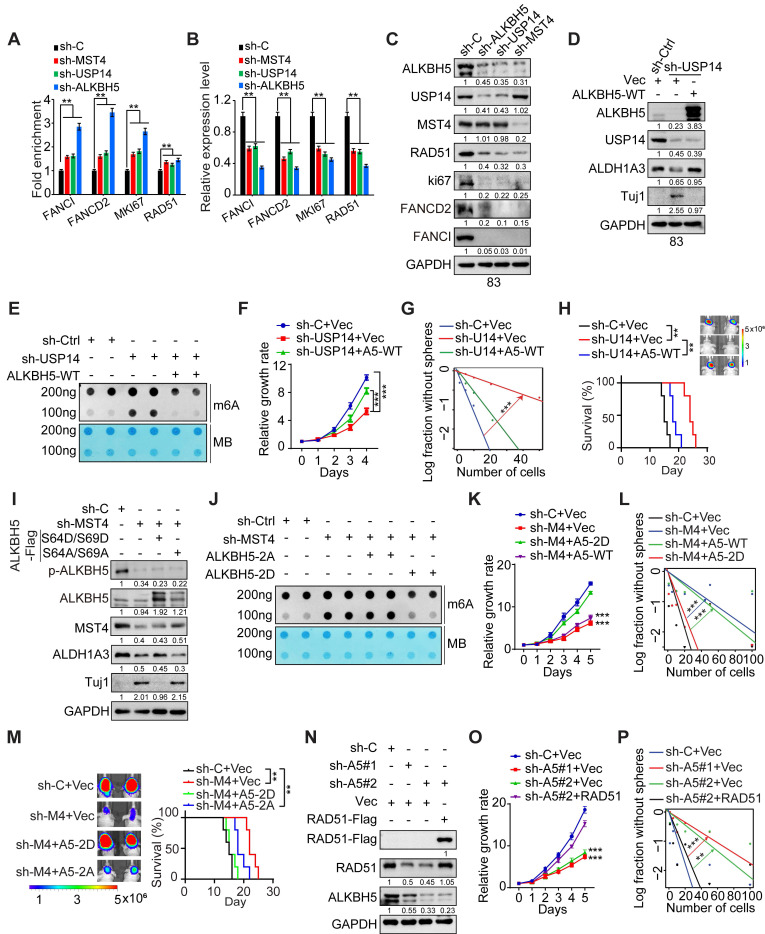
** MST4-USP14-ALKBH5 signaling pathway modulates the self-renewal capacity, DNA repair mechanisms, and tumorigenic potential of GSCs.** (**A-C**) MeRIP-qPCR (A), quantitative PCR (B), and IB (C) analyses of FANCI, FANCD2, MKI67, and RAD51 expression levels in GSC83 cells after the depletion of MST4, USP14, or ALKBH5. (**D**) IB analysis of indicated proteins in GSC83 cells with or without USP14 knockdown or expression of ALKBH5-WT. (**E** and** J**) Levels of the m^6^A RNA modification were assessed via RNA dot blot in GSC83 cells with indicated modifications. Methylene blue detected RNA loading. (**F** and **G**) Cell proliferation (F) or limiting dilution neurosphere-forming assay (G) using GSC 83 cells with indicated modifications. (**H** and** M**) *In vivo* BLI and Kaplan-Meier survival analysis of mice intracranially injected with the indicated GSC 83 (n = 5/group, left). (**I** and** N**) IB analysis of indicated proteins in GSC 83 cells with indicated modifications. (**K** and **L**) Effects of MST4 knockdown, with or without reconstituted expression of ALKBH5-WT or 2D, on cell proliferation (K) and sphere-forming frequency (L) of GSC 83 cells. (**O** and **P**) Cell proliferation (O) and sphere-forming frequency (P) of GSC 83 cells expressing sh-C or sh-ALKBH5, with or without overexpression of RAD51. Statistical significance was determined by one-way ANOVA with Tukey's multiple comparisons test (A and B), two-way ANOVA test (F, K and O), the likelihood ratio test (G, L and P) and the log-rank test (H and M). Data are representative of two to three independent experiments with similar results. All bar graph data are presented as means ± SEM. **p < 0.01, ***p < 0.001.

Cytotoxic therapies for GBM, including IR and TMZ, have largely failed due to the tumor cells' strong DNA repair abilities, particularly in those with stem cell-like traits. Double-strand breaks (DSBs) represent the most severe type of DNA damage, and DSBR that enables cell survival contributes to therapeutic resistance and treatment failure 40. Notably, many of the DSBR-associated genes involved in HR, such as *FANCI*, *FANCD2*, *MKI67*, and *RAD51* 14. KD of ALKBH5, MST4 or USP14 increased the m^6^A enrichment on transcripts of *FANCI*, *FANCD2*, *MKI67*, and *RAD51*, resulting in decreased their mRNA and protein levels (Figure 6A-C). Remarkably, our results demonstrated that the KD of ALKBH5 resulted in an increase in m^6^A methylation levels within the 5' untranslated region (UTR) of *USP14* mRNA, alongside a notable decrease in both mRNA and protein expression levels of USP14 (Figure 6C and S6H-K). These alterations were reversed upon restoration of ALKBH5 expression (Figure S6I-K). Subsequently, we investigated the direct interaction between ALKBH5 and *USP14* mRNA by conducting ALKBH5 RNA immunoprecipitation (RIP) followed by PCR analysis. The results demonstrated that ALKBH5 RIP substantially enriched *USP14* mRNA within the m^6^A modification region, indicating that ALKBH5 associates with *USP14* mRNA in an m^6^A-dependent manner (Figure S6L). To investigate the role of ALKBH5 in modulating *USP14* mRNA levels via its stability, we treated GSCs with the transcription inhibitor actinomycin D (Act D) and observed that ALKBH5 KD significantly reduced the half-life of *USP14* transcripts (Figure S6M). Additionally, the KD of YTHDF2, in contrast to YTHDF1 or YTHDF3, reinstated *USP14* mRNA levels in ALKBH5-deficient GSCs (Figure S6N), suggesting that YTHDF2 acts as the m^6^A reader for *USP14* mRNA. Collectively, these findings indicate that ALKBH5 modulates *USP14* mRNA stability in GSCs through an m^6^A-dependent mechanism involving YTHDF2.

To investigate the significance of USP14-mediated deubiquitylation of ALKBH5 in the context of USP14-enhanced GSC tumorigenicity, we reintroduced exogenous ALKBH5-WT into GSC83 cells where endogenous USP14 was knocked down (Figure 6D). The KD of USP14 resulted in elevated global levels of m^6^A (Figure 6E) and inhibited both the proliferation and sphere-forming capacity of GSCs *in vitro* (Figure 6F-G). Furthermore, it significantly suppressed the growth of intracranial tumor xenografts in athymic nude mice, leading to a notable extension of survival in the animal subjects (Figure 6H). The exogenous expression of ALKBH5 effectively rescued the impacts of USP14 KD on RNA m^6^A methylation, GSC proliferation, sphere-forming frequency *in vitro*, and the tumorigenicity of orthotopic glioblastoma xenografts (Figure 6E-H).

To evaluate the significance of MST4-mediated phosphorylation at S64/S69 of ALKBH5 in the context of MST4-augmented GSC tumorigenicity, we introduced exogenous phosphomimic mutants S64D/S69D and non-phosphorylatable mutants S64A/S69A of ALKBH5 into GSC83 cells, which had undergone depletion of endogenous MST4 (Figure 6I). The KD of MST4 was associated with an elevation in global m^6^A levels (Figure 6J), a reduction in GSC proliferation and sphere-forming capacity (Figure 6K-L), as well as decreased growth of intracranial tumor xenografts in athymic nude mice, evidenced by extended survival of the animals (Figure 6M). The ALKBH5 S64D/S69D mutant, in contrast to S64A/S69A, effectively mitigated the inhibitory impact of MST4 KD on m^6^A demethylation, GSC proliferation, sphere-forming ability *in vitro*, and tumorigenic potential *in vivo* (Figure 6J-M).

GSCs exhibit resistance to IR therapy that causes DNA damage 41. Subsequently, we explore the involvement of the MST4-USP14-ALKBH5 signaling cascade in the DNA damage repair mechanisms within GSCs. Although KD of MST4, USP14, or ALKBH5 did not alter the formation of phospho-Histone 2A.X (γH2AX) foci, an indicator of DNA double-strand breaks (DSBs), the proportion of γH2AX foci-positive cells in these GSCs was markedly elevated compared to control cells 4 h post-IR treatment (Figure S7A-E), indicating impaired DSBR in MST4, USP14, or ALKBH5-deficient cells. The reintroduction of ALKBH5 significantly mitigated the impact of USP14 KD on GSC sensitivity to IR, as evidenced by reduced DNA damage and enhanced cell survival following IR exposure (Figure S7F-G).

Radiation-induced DSBs are primarily repaired via homologous recombination (HR) and non-homologous end joining (NHEJ). To assess whether the MST4-USP14-ALKBH5 signaling axis facilitates either of these repair pathways, or both, a specific inhibitor for HR and NHEJ was employed. In the presence of B02, an inhibitor that specifically targets RAD51 and the HR pathway, the overexpression of MST4, USP14, or wild-type ALKBH5, unlike the ALKBH5-S64A/S69A variant, did not significantly increase the repair efficiency of DSBs induced by IR (Figure S7H-J). NU7441, a selective inhibitor of DNA-PK within the NHEJ pathway, did not alter the efficiency of DSB-DNA repair that was enhanced by MST4, USP14, or ALKBH5-WT (Figure S7H-J), suggesting that the MST4-USP14-ALKBH5 signaling axis specifically regulates HR rather than NHEJ pathway.

Given that the pharmacological inhibition of RAD51 interferes with the MST4-USP14-ALKBH5 signaling axis involved in DNA damage repair, we aim to determine whether RAD51 serves as a principal downstream effector of the MST4-USP14-ALKBH5 signaling pathway in the context of DNA repair within GSCs. RAD51 exhibited a positive correlation with the expression levels of ALKBH5 and USP14 in GBM tumors according to the CGGA dataset (Figure S8A). Elevated RAD51 expression correlated with tumor progression and a poorer prognosis in both TCGA and CGGA datasets (Figure S8B-F). Furthermore, ALKBH5 directly interacted with *RAD51* mRNA (Figure S6L), and the KD of ALKBH5 reduced the half-life of *RAD51* transcripts (Figure S6M). Additionally, KD of YTHDF2 could restore *RAD51* mRNA levels in ALKBH5-depleted GSCs (Figure S6N), suggesting that ALKBH5 regulates the m^6^A modification and stability of *RAD51* mRNA via YTHDF2. Notably, overexpression of exogenous RAD51 significantly mitigated the inhibitory effects of ALKBH5 KD on GSC proliferation and sphere-forming capacity *in vitro*, along with enhancing resistance to IR treatment (Figure 6N-P and S8G).

### Inhibition of USP14 reduces GSC tumorigenesis and increases GBM sensitivity to IR

**Figure 7:**
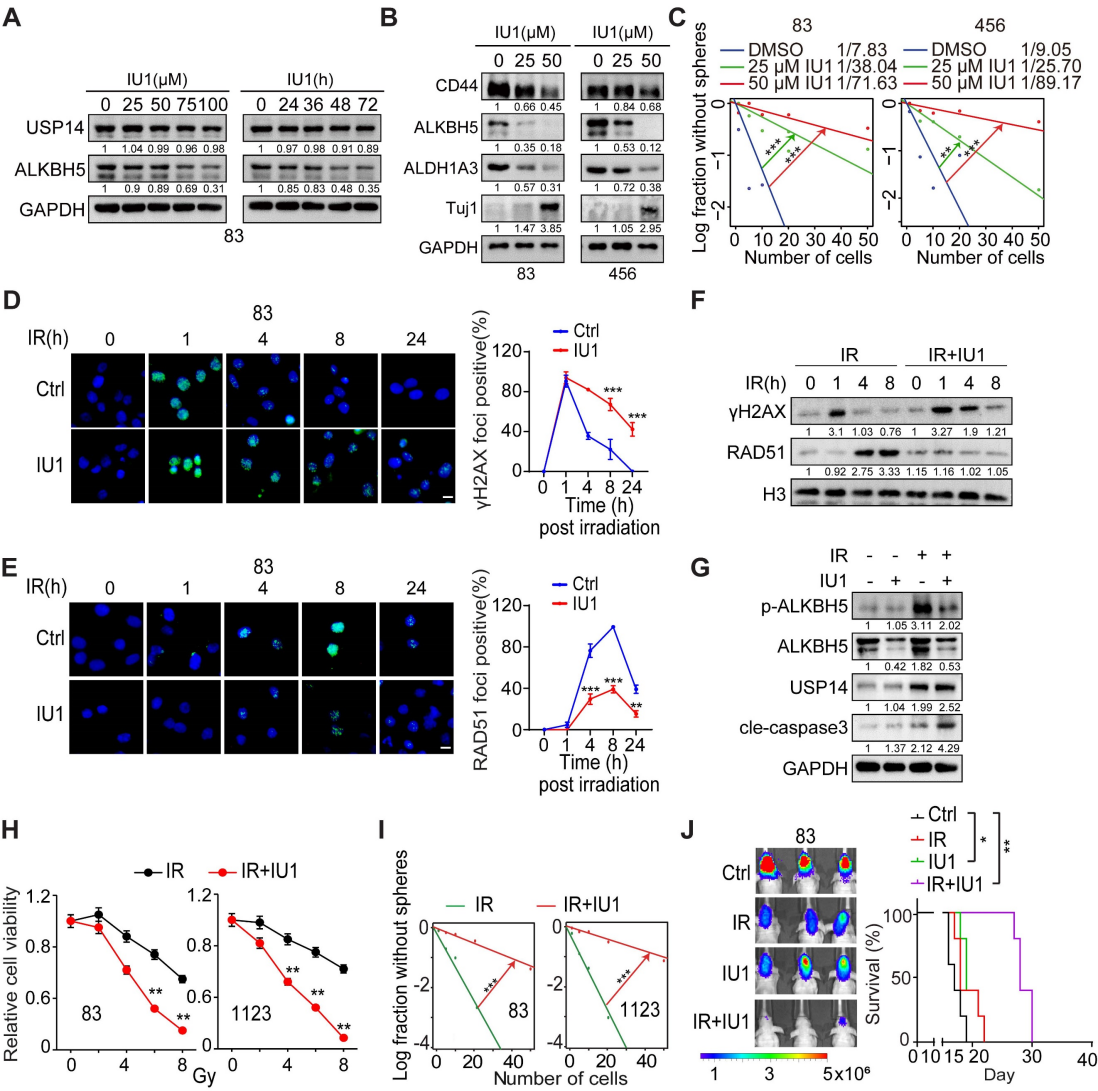
** Inhibiting USP14 with IU1 reduces the tumor-initiating capacity of GSCs and makes GSC tumor xenografts more responsive to IR.** (**A**) IB analysis of indicated proteins in GSC 83 cells that were treated with the indicated concentrations of IU1 for 24 h (left) or with 20 μM IU1 for the indicated time (right). (**B**) IB analysis of indicated proteins in GSC 83 and 456 cells that were treated with the indicated concentrations of IU1 for 48 h. (**C**) Sphere-forming frequency for GSC 83 cells treated with the indicated concentrations of IU1 for 48 h. (**D** and **E**) Representative images (left) and quantification data (right) for γH2AX (D) and RAD51 (E) formation in GSC 83 cells that were exposed to 5 Gy of IR and 25 μM of IU1. Cells were subsequently stained at indicated time points post-IR with antibodies against γH2AX or RAD51. Cells exhibiting more than eight foci for γH2AX or over five foci for RAD51 were considered positive. Scale bar = 10 μm. (**F**) IB of indicated proteins in GSC 83 cells treated with IR (5 Gy), or IU1 (25 μM) + IR. (**G**) IB of indicated proteins in GSC 83 cells at day 2 after indicated treatments. IR, 5 Gy; IU1, 25 μM. (**H** and **I**) Cell viability (H) and sphere formation frequency (I) for GSC 83 and 1123 cells on day 5 after the indicated treatments. (**J**) BLI images and Kaplan-Meier analysis of GBM brain xenografts of GSC 83 with indicated treatments (n = 5/group). Colored scale bars represent photons/s/cm^2^/steradian. Statistical significance was determined by the likelihood ratio test (C and I), two-tailed Student's t-test (D, E and H) and the log-rank test (J). The data presented in the bar or line graphs are expressed as mean ± SEM. *p < 0.05, **p < 0.01, and ***p < 0.001. Data are representative of two to three independent experiments with similar results.

Subsequently, we evaluated the impact of the selective USP14 inhibitor IU1, which has demonstrated *in vivo* bioavailability 42, 43, on the tumorigenic potential of GSCs. IU1 was found to reduce the levels of ALKBH5 in a dose- and time-dependent manner, thereby corroborating the observations that USP14 plays a role in the stabilization of ALKBH5 (Figure 7A). Additionally, IU1 was found to downregulate the expression of ALDH1A3 while simultaneously upregulating Tuj1, leading to a reduction in GSC properties (Figure 7B-C and S9A). Importantly, IU1 demonstrated no cytotoxic effects on normal neural progenitor cells (NPCs) (Figure S9B). The DSB marker γH2AX was acutely induced in cells exposed to IR, but in GSCs treated with the USP14 inhibitor IU1, γH2AX levels remained elevated for 24 h post-IR exposure, indicating the persistence of unresolved DSBs (Figure 7D and S9C). Moreover, treatment with IU1 significantly diminished the recruitment of RAD51 to sites of DNA damage following IR when compared to control cells (Figure 7E). Consistent with this, immunoblot analyses assessing the kinetics of γH2AX and RAD51 after IR revealed that γH2AX levels were comparable between the IR and IR+IU1 conditions at 1 h post-IR. However, starting at 4 h post-IR, γH2AX levels in IR+IU1 cells were markedly elevated compared to those in IR cells, coinciding with a reduction in RAD51 chromatin association following IR exposure (Figure 7F). IR exposure resulted in increased expression of ALKBH5 and USP14, along with enhanced phosphorylation of ALKBH5 in GSCs (Figure 7G). The inhibition of USP14 by IU1 abrogated the IR-induced upregulation of ALKBH5 and sensitized GSCs to IR by promoting apoptotic pathways (Figure 7G). These molecular alterations were paralleled by a reduction in cell viability and sphere-forming capacity following the combination treatment, in contrast to treatment with IR alone (Figure 7H-I). The *in vivo* co-administration of IU1 in athymic nude mice bearing brain tumor xenografts significantly amplified the cytotoxic effects of IR, as evidenced by a notable increase in survival rates compared to those receiving monotherapy (either IU1 or radiotherapy) (Figure 7J). Xenografts treated with IU1 exhibited diminished expression levels of Ki67 (a marker of cell proliferation) and RAD51 (an indicator of HR), alongside heightened m^6^A staining (Figure S9D-E). This suggests that IU1 effectively inhibits ALKBH5 activity and DNA repair mechanisms within the xenografts. While IR promoted the phosphorylation of ALKBH5, the combination of IU1 with IR (IU+IR) resulted in elevated levels of γH2AX (indicative of DSBs) and increased apoptosis (as shown by cleaved caspase-3) in the treated tumors (Figure S9D-E). These findings indicate that IU1 significantly enhances the cytotoxic response of IR against the progression of intracranial tumors compared to monotherapies.

### The co-expression of MST4, p-ALKBH5, and USP14 serves as a prognostic indicator for clinical GBM

**Figure 8:**
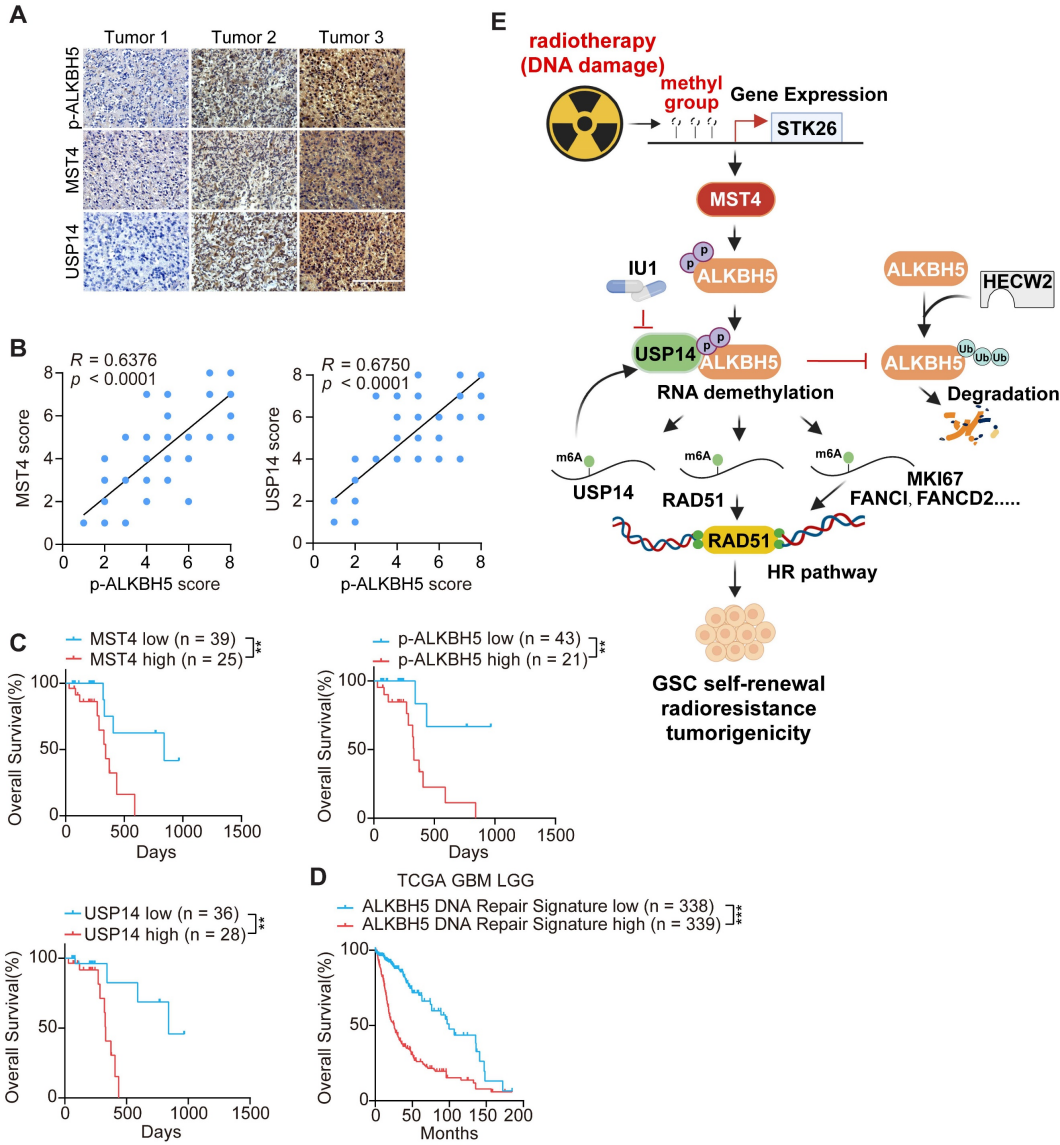
** Correlative expressions of MST4, p-ALKBH5, and USP14 are prognostic markers for clinical GBM.** (**A**) Representative images of IHC staining of MST4, p-ALKBH5, and USP14 in clinical GBM samples. Scale bars = 200 μm. (**B**) Correlations of IHC with MST4 (left), USP14 (right) expression relative to the level of p-ALKBH5. (**C**) Kaplan-Meier analyses for GBM patients with tumors expression high or low level of MST4, p-ALKBH5, or USP14. (**D**) Kaplan-Meier survival analyses of TCGA glioma patients with high or low levels of ALKBH5-regulated DNA damage gene signature. (**E**) Illustration of the MST4-USP14-ALKBH5 signaling pathway in regulation of RNA m^6^A methylation, HR-DSBR, tumorigenicity, and radiation resistance of GSCs. Statistical significance was determined by Pearson correlation test (B), or log-rank test (C, D). **p < 0.01, ***p < 0.001.

To further elucidate the significance of MST4, USP14, and p-ALKBH5 in the biological behavior of GBM, we conducted an immunohistochemical (IHC) analysis of their expression profiles in 64 de-identified glioma patient samples (Figure 8A and Table S1). MST4 and USP14 staining showed positive association with the p-ALKBH5 results (Figure 8B). The clinical relevance of these findings was underscored by the observation that patient survival exhibited an inverse correlation with the staining levels of MST4, USP14, and p-ALKBH5 (Figure 8C). To further substantiate the clinical importance of the MST4-USP14-ALKBH5 signaling axis in the context of IR resistance, a DNA repair gene signature characterized by the downregulation of genes following ALKBH5 KD was found to correlate with reduced patient survival in the TCGA glioma cohort (Figure 8D). Additionally, the mRNA expression levels of MST4/STK26, USP14, and ALKBH5 were positively correlated with ALKBH5 target genes, including FANCD2, FANCI, MKI67, and RAD51 within the TCGA and CGGA glioma datasets (Figure S10A-B).

## Discussion

This study identifies a MST4-USP14-ALKBH5 signaling axis that plays a crucial role in enhancing the stem-like properties, DNA repair capabilities, radioresistance, and tumorigenic potential of GSCs. The cascade is triggered by the phosphorylation of ALKBH5 at serine residues S64 and S69 by MST4, which in turn inhibits the ubiquitination of ALKBH5 via the E3 ubiquitin ligase HECW2. This mechanism promotes the interaction and deubiquitylation of ALKBH5 by USP14, leading to an increase in ALKBH5 levels within GSCs. Importantly, ALKBH5 directly binds to and stabilizes the USP14 mRNA in a YTHDF2-dependent manner, thereby creating a positive feedback loop that sustains the overexpression of both USP14 and ALKBH5 in GSCs. Additionally, a signature indicative of DSBR activation is markedly enriched among the downstream targets of ALKBH5. The MST4-USP14-ALKBH5 pathway diminishes m^6^A methylation on homologous recombination (HR)-related transcripts such as FANCI, FANCD2, MKI67, and RAD51, thereby bolstering resistance to IR. Furthermore, this signaling pathway is activated upon exposure to IR. These observations clarify the reduced m^6^A levels observed in GSC mRNA following IR treatment. The application of IU1, a specific inhibitor of USP14, to disrupt USP14-mediated stabilization of ALKBH5 in GSCs enhances the cytotoxic effects of IR on GBM by compromising HR-mediated DNA repair. Increased levels of phosphorylated ALKBH5 correlate with unfavorable clinical outcomes in glioma patients. Our results suggest that targeting the MST4-USP14-ALKBH5 signaling axis may provide a promising therapeutic strategy for the treatment of GSCs.

Aberrant expression of USP14 is associated with progression of various cancers, including colorectal carcinoma 26, hepatocellular carcinoma 44, pancreatic ductal adenocarcinoma 27, breast cancer 45, and gastric cancer 46. Nevertheless, the role of USP14 in glioma remains to be elucidated. In this study, we demonstrate that USP14 is markedly upregulated in MES GSCs compared to PN GSCs. The deubiquitinating enzymatic activity of USP14 is crucial for preserving stem-like properties, radioresistance, and tumorigenicity of GSCs. USP14 interacts with and deubiquitinates the m^6^A demethylase ALKBH5, thereby promoting the stabilization and elevated expression of ALKBH5 within GSCs. Additionally, the upregulation of USP14 following IR is associated with ALKBH5-mediated demethylation of m^6^A modifications on *USP14* mRNA, enhancing its stability. IU1, a selective small-molecule inhibitor of USP14 43, significantly reduces ALKBH5 protein levels and disrupts HR- DSBR as well as radioresistance in GSCs. The combination of IU1 with radiotherapy inhibits the proliferation of intracranial GBM xenografts, leading to a substantial extension of survival in the treated murine models. Our findings reveal a novel role of USP14 in the regulation of RNA m^6^A modifications and the pathophysiological mechanisms of GBM, suggesting that USP14 may serve as a promising therapeutic target for GBM management.

Increasing evidence has revealed an association between the transcriptional expression levels of ALKBH5 in tumor cells and poor clinical outcomes in GBM patients, indicating ALKBH5 as a potential therapeutic target in GBM 14, 47, 48. However, according to Protein Atlas, the protein levels of ALKBH5 do not show substantial differences between normal brain tissues and GBM samples (https://www.proteinatlas.org/ENSG00000091542-ALKBH5/pathology/glioma), suggesting that post-translational modifications, such as ubiquitination, may influence the cellular levels of ALKBH5 protein. Furthermore, ALKBH5 is aberrantly expressed in various cancers, where its expression can either promote or inhibit tumorigenesis depending on the cancer type 49. Given the dysregulation and essential roles of ALKBH5 in maintaining CSCs across various tumors, including breast cancer 16, 49, GBM 14, acute myeloid leukemia 15, and endometrial carcinoma 50, ALKBH5 is emerging as a promising target for therapeutic strategies aimed at eradicating CSCs and achieving complete remission in cancer therapies. Specifically, hypoxia-induced upregulation of ALKBH5 promotes the stabilization of pluripotency factor mRNAs by reducing m^6^A methylation, thereby facilitating the maintenance of breast CSCs 16. In acute myeloid leukemia (AML), the overexpression of ALKBH5 facilitates the self-renewal of leukemia stem/initiating cells (LSCs/LICs) by enhancing the expression of TACC3 15. Although prior research has established that ALKBH5 is essential for the self-renewal of GSCs 14, the interplay between ALKBH5 and DNA repair remains insufficiently understood. This study demonstrates that ALKBH5 protein levels, rather than mRNA expression, are significantly elevated in GSCs post-IR treatment, indicating that post-translational modifications may critically govern ALKBH5 expression under certain conditions. Alongside a reduction in the ubiquitylation of ALKBH5 protein, its phosphorylation is also induced in GSCs following IR. MST4 serves as the upstream kinase that phosphorylates ALKBH5 in response to IR, facilitating the subsequent binding and deubiquitylation of ALKBH5 by USP14, ultimately contributing to the stabilization and upregulation of the ALKBH5 protein. The transcripts that exhibit differential expression or methylation upon ALKBH5 depletion are enriched for genes involved in DNA repair, indicating that ALKBH5 modulates m^6^A methylation on critical DSBR-related transcripts, such as FANCI, FANCD2, MKI67, and RAD51, thereby enhancing their expression. These findings elucidate the mechanisms underlying the accelerated DNA repair processes and increased therapeutic resistance observed in GSCs. Consequently, manipulating the phosphorylation and ubiquitination status of ALKBH5 may represent a viable post-translational strategy to attenuate the activity and functionality of ALKBH5.

In conclusion, we have identified a MST4-USP14-ALKBH5 signaling pathway that plays a key role in the DNA repair processes, stem-like traits, and treatment resistance in GSCs. Our findings show that the USP14 inhibitor IU1 significantly increases the sensitivity of GSC-derived brain tumor xenografts to IR therapy, supporting the idea of targeting USP14 to enhance standard GBM treatment strategies.

## Materials and Methods

### Ethics statement

This study follows ethical regulations. Experiments using patient specimens were approved by the institutional review boards of Union Hospital, Tongji Medical College, Huazhong University of Science and Technology (UHHUST) with informed consent from the donors, and conducted in accordance with Xiamen University. Samples were acquired as part of the UHHUST institutional review board-approved clinical protocol no. UHCT-IEC-SOP-016-03-01. All mouse experiments were completed in accordance with the Guidelines for the Care and Use of Laboratory Animals and were approved by the Institutional Animal Care and Use Committee (IACUC) at Xiamen University. Experiments were performed in accordance with a protocol approved by the Xiamen University (no. XMULAC20220136).

### Xenograft studies

Due to evidence showing that male patients have a 1.6 times higher incidence and poorer prognosis after treatment than females 51, male athymic mice, aged 4-5 weeks, were obtained from Guangdong Yaokang Biotechnology Co., Ltd. for intracranial injection in this study. Each cage contained five mice. GSC83 cells were established from a 72-year-old patient diagnosed with GBM, and their stemness characteristics were confirmed as previously described 31. GSCs were dissociated into a single-cell suspension utilizing StemPro™ accutase. The viable cell concentration was assessed by enumerating Trypan Blue exclusion-negative cells using a hemocytometer. The nude mice were anesthetized using isoflurane inhalation and immobilized. The head skin was disinfected with an alcohol-soaked cotton pad, and the periosteum was removed to expose the anterior fontanelle. GSC spheres (5 x 10^4^ in 5 µL PBS) with indicated expression constructs were intracranially injected into the brains of individual mice using the following coordinates from bregma: 2.5 mm lateral, 1.5 mm anterior, and 3.0 mm deep from the skull.

Bioluminescence imaging was performed to monitor *in vivo* tumor growth using the IVIS Lumina imaging station (Caliper Life Sciences). Tumor-bearing mice were injected 300 mg/kg of D-luciferin (potassium salt, Gold Biotechnology) before isoflurane anesthesia. Daily monitoring of mouse behavior and body weight was conducted. Upon manifestation of symptoms such as hunched back, unstable gait, weight loss exceeding 10%, or leg paralysis, mice were euthanized and perfused for subsequent collection and embedding of brain tissues in OCT compound followed by freezing conservation. Survival curves between groups were compared using the log-rank test. Tissue sections (10 µm thick) were obtained using a Leica frozen microtome, with subsequent hematoxylin and eosin staining or immunostaining performed on brain tumor sections. Each measurement was validated through IHC analysis of protein expression in tumor tissues.

### Cell lines and cell culture

Human HEK293T cells (ATCC) were cultured in DMEM (Invitrogen) supplemented with 10% FBS and 1% penicillin and streptomycin. Patient-derived glioma stem-like cells (GSCs), which were previously characterized 31, 52-54, were cultured as non-adherent spheroids in DMEM/F12 medium (Thermo Fisher Scientific), supplemented with B27 (2%, Thermo Fisher Scientific), penicillin and streptomycin (1%, Thermo Fisher Scientific), Heparin (5 μg/mL, Sigma-Aldrich), EGF (20 ng/mL, Peprotech), and bFGF (20 ng/mL, Peprotech). Normal human astrocyte (Thermo Fisher Scientific, N7805100) cells were cultured in the astrocyte growth medium (Lonza, CC-3187).

### Antibodies and reagents

The antibodies and reagents used in this study including Anti-USP14 (ABclonal, Cat# A16643; RRID: AB_2772821, 1:1000); Anti-GAPDH (Proteintech, Cat# 60004-1-Ig; RRID: AB_2107436, 1:10000); Anti-CD44 (Santa Cruz Biotechnology, Cat# sc-7297; RRID: AB_627065, 1:1000); Anti-ALDH1A3 (Santa Cruz Biotechnology, Cat# sc-376089; RRID: AB_10988564, 1:500); Anti-ALKBH5 (ABclonal, Cat#A11684; RRID: AB_2758686); Anti-Flag (Shanghai Genomics Technology, Cat# GNI 4110-FG; RRID: AB_3067995, 1:1000); Anti-Myc (Proteintech, Cat# 16286-1-AP; RRID: AB_11182162, 1:1000); Anti-STK26/MST4 (ABclonal, Cat# A16534; RRID: AB_2772436, 1:1000); Anti-MST4 (Proteintech, Cat# 10847-1-AP; RRID: AB_513907, 1:1000); Anti-m^6^A (ABclonal, Cat# A19841; RRID: AB_2862753, 1:500); Anti-m^6^A (Synaptic Systems, Cat# 202003; RRID: AB_2279214, 1:1000); Anti-RAD51 (Santa Cruz Biotechnology, Cat# sc-377467; RRID: AB_2910142, 1:1000); Anti-Tuj1 (Santa Cruz Biotechnology, Cat# sc-80005; RRID: AB_2210816, 1:500); Anti-pH2A.X (Santa Cruz Biotechnology, Cat# sc-517348; RRID: AB_2783871, 1:1000); Anti-HA (Proteintech, Cat# HRP-81290; RRID: AB_3086578, 1:1000); Anti-HA (ABclonal, Cat# AE008; RRID: AB_2770404, 1:1000); Anti-SOX2 (Proteintech, Cat# 66411-1-Ig; RRID: AB_2881783, 1:1000); Anti-β-actin (ABclonal, Cat# AC026; RRID: AB_2768234, 1:10000); Anti-p-Ser/Thr (ECM Biosciences, Cat# PP2551; RRID: AB_1184778, 1:1000); Anti-p-Tyr (ECM Biosciences, Cat# PP2221; RRID: AB_715277, 1:1000); Anti-KI67 (Proteintech, Cat# 27309-1-AP; RRID: AB_2756525, 1:1000). A mouse polyclonal antibody targeting phospho-ALKBH5 was produced through the immunization of subjects with a synthetic phosphopeptide corresponding to the residues surrounding S64/S69 of human ALKBH5, followed by affinity purification. Nonspecific IgGs served as negative controls. HRP-conjugated goat anti-rabbit IgG (H+L) (ABclonal, Cat# AS014; RRID: AB_2769854, 1:10000); HRP-conjugated goat anti-mouse IgG (H+L) (ImmunoWay, Cat# RS0001; RRID: AB_2943495, 1:10000); VeriBlot for IP Detection (HRP) (Abcam, Cat# ab131366; 1:2000).

### Immunoblot analysis (IB)

Cells were lysed in a RIPA buffer (50 mM Tris-HCl, pH 8.0;150 mM Sodium chloride;1% NP-40; 0.5% sodium deoxycholate; 0.1% sodium dodecyl sulfate; 2 mM EDTA) containing 1 × protease and 1× phosphotase inhibitor cocktails (MCE). Protein samples were quantified by using the Bradford assay reagent. Protein samples were subjected to boiling at 99 °C for 10 min, low speed centrifugation, SDS-PAGE and transferred to polyvinylidene fluoride (PVDF, Millipore) membranes. Membranes were incubated with indicated antibodies for overnight at 4 °C. Following wash with TBS-T (TBS containing 0.1% Tween-20), the blot was incubated with corresponding peroxidase-labeled secondary antibodies. Blots were developed with enhanced chemiluminescence (ECL, Amersham Bioscience) reaction according to manufacturer instructions.

### Plasmid construction and construction of stable cell lines

shRNA oligos targeting USP14, ALKBH5, MST4 and YTHDF1/2/3 were constructed into the pLKO.1 lentivirus vector according to a standard protocol provided by Addgene (https://www.addgene.org/protocols/plko/). MST4, USP14, ALKBH5, RAD51 ORFs (open reading frames) were cloned into the pCDH-CMV-MCS-EF1-Puro or pLVX-puro vector to generate corresponding gene expression plasmids. Site-directed mutagenesis was performed with a QuikChange mutagenesis kit (Agilent Technologies), according to the manufacturer's instructions. The sgRNA sequence of the target gene was designed using the CRISPR design program (http://tools.genome-engineering.org/). The synthesized forward and reverse primers including 20 bp target sequence and BsmBI sticky end were annealed and inserted into the lentiCRISPRv2GFP digested with BsmBI.

Lentiviral constructs expressing shRNAs, or full-length MST4, USP14, ALKBH5, RAD51 or their mutant cDNAs were transfected into HEK293T using Lipofectamine 2000 (Thermo Fisher Scientific, Cat# 11668027) in accordance to the manufacturer's instructions. To establish stable cell lines, the supernatants containing lentivirus were harvested 48-72 h after transfection and used to infect target cells with polybrene. After 72 h of transduction, cells were selected with 2.0 μg/mL puromycin for 4 days.

### Cell proliferation, viability, and limited dilution assays

GSC spheres were dissociated into single cells, and cell density was quantified by counting viable (Trypan Blue negative) cells using a hematocytometer. The number of living cells was determined at different time points using a hematocytometer. Cells were seed into a 24-well plate containing 1 mL culture medium at a density of 5000 cells per well. Cell viabilities were evaluated using CellTiter-Glo® 2.0 Cell Viability Assay (Promega) according to the manufacturer's instructions. The dissociated GSCs seeded in 96-well plates at density of 1, 5, 10, 20 or 50 (n = 24 in each dilution) for GSC cells. After 7 days, each well was examined for formation of tumor spheres. Extreme limiting dilution assays/sphere forming frequency were analyzed using a software available at http://bioinf.wehi.edu.au/software/elda/.

### Immunofluorescent staining

GSCs and frozen brain tissue sections were fixed with 4% PFA for 10 min and then permeabilization with 0.1% Triton X-100, and blocked with 5%BSA for 60 min and then incubated with following primary antibodies 4 °C overnight. After being washed three times with PBS, cells were incubated with Alexa 488 and 594 labeled secondary antibodies (1:500) and DAPI-containing mounting solution Vectashield (Vector Laboratories) for 1 h, and then visualized by using a Nikon inverted microscope Eclipse Ti-U equipped with a digital camera.

### Immunoprecipitation (IP)

Cells were lysed with IP lysis buffer (Genstar) supplemented with proteinase inhibitor and phosphatase inhibitor cocktail (MCE), incubated on ice for 15 min, and cleared by centrifugation at 13,000 g at 4 °C for 15 min. The cell lysates were incubated overnight with the corresponding antibodies and protein G agarose beads, followed by washing, elution, and detection.

### Dot blot assay

Total RNA was extracted from GSCs using the miRNeasy Mini kit (QIAGEN). Subsequently, mRNA purification was performed by employing Oligo (dT) - 25 Dynabeads (Invitrogen). Denaturation of mRNA occurred at 95 °C for 3 min, followed by immediate immersion in an ice bath for 2 min. After quantification, RNA samples were spotted on Hybond-N+ membranes (Amersham) and cross-linked with violet light (1200 microjoules [x100]; 25-50 s). Unbound mRNA was washed off with PBS for 5 min at room temperature. Following blocking with the 5% skim milk buffer for 1-2 h, cells were incubated overnight at 4 °C with anti-m^6^A antibody (Synaptic Systems). After three washes with TBS-T buffer, HRP-coupled sheep rabbit IgG resistance was used to incubate the samples at room temperature for 1 h. Finally, imaging was conducted after washing through TBST buffer.

### Proteomics analysis

Proteomics analysis for USP14-interacted proteins and ALKBH5 phosphorylation in GSCs was performed at the core facility of biomedical sciences, Xiamen University. The analysis for protein phosphorylation as previous described 30.

### RNA pulldown

Biotin-labeled USP14, RAD51 mRNAs were incubated with M-280 Streptavidin Dynabeads (Invitrogen) for 30 min. Subsequently, washing was performed using NT2 buffer (50 mM Tris-HCl [pH 7.4], 150 mM NaCl, 1 mM MgCl_2_, and 0.05% NP-40). The beads were then subjected to the incubation at 4 °C for 2 h with FLAG-ALKBH5-expressing cell lysates. After a series of washes using high-salt NT2 buffer, RNA-binding proteins were eluted utilizing a solution containing 2 mM D-biotin.

### Quantitative analysis of m^6^A modification of RNA by LC-MS

The m^6^A modification of RNA was quantified using liquid chromatography-mass spectrometry (LC-MS), following the methodology described in previous studies 55. Total RNA was extracted using Oligo (dT) - 25 Dynabeads (Invitrogen) purification, followed by mRNA purification using a nucleoside enzyme mixture (#M0649S, NEB). N^6^-methyl adenosine and adenosine were analyzed using Q Exactive Mass Spectrometers (Thermo Fisher).

### MeRIP

The MeRIP assay was conducted using the Magna MeRIP m^6^A kit (#17-1094, Millipore Sigma). RNA was denatured by repeated heating at 95 °C for 5 min followed by vertexing. Subsequently, RNA precipitation was performed with ethanol and incubated overnight at 80 °C, dried, and resuspended in RNase-free water. Immunoprecipitation was carried out using Magna ChIP Protein A/G magnetic beads (#17-10085, Sigma-Aldrich) coupled with 10 μg of anti-m^6^A antibody and 300 μg of RNA. After a 2 h incubation at 4 °C, the beads were washed and the eluted RNA was subjected to purification using the RNeasy mini kit (Qiagen). Following delivery and quality control assessment, sequencing or qPCR analysis was performed. Following UV crosslinking, GSC samples underwent lysis with lysates supplemented with protease inhibitors and ribonuclease inhibitors. A fraction constituting 5% of the cell lysis solution served as the input sample for subsequent analysis while total RNA extraction employed TRIzol reagent. The remaining 95% of cell lysates underwent IP experiments utilizing anti-flag M2 Magnetic Beads (#M8823, Sigma-Aldrich), followed by digestion with proteinase K and subsequent RNA component extraction employing TRIzol. The cDNA was synthesized through reverse transcription using the SuperScript III first-strand synthesis kit (Invitrogen). Real-time PCR reactions were also conducted using the SYBR Select Master Mix (Applied Biosystems).

### RNA decay assay

Cells were collected after the treatment of actinomycin D at a final concentration of 5 μg/mL for indicated time. Total RNA was prepared by TRIzol and subject to RT-PCR analysis. GAPDH was used as endogenous control. The half-life of mRNA was measured according to a previous study 56.

### RIP-PCR

After crosslinked by UV (stratalinker 1800 (254 nm), 400 mJ/cm^2^), the nuclear fragment of cells was extracted and sonicated. 2 μg of specific antibody for ALKBH5 or a negative control rabbit IgG was conjugated to protein A/G magnetic beads (Thermo Fisher Scientific), and incubated with pre-cleared nuclear extraction at 4 °C for overnight. After washing with RIP buffer (25 mM Tris-HCl, 5 mM EDTA, 0.5 mM DTT, 150 mM KCl, 0.5% NP40, 1× protease inhibitor, 1× RNase inhibitor) for five times. Beads were resuspended with PBS and removed DNA contamination with DNase and proteinase K. The RNA from input and co-immunoprecipitation with antibodies were extract by TRIzol (Invitrogen) and analyzed by quantitative PCR.

### Quantitative RT-PCR (qPCR)

Total RNA was extracted using Trizol reagent (Invitrogen), and cDNA was prepared using the PrimeScript cDNA synthesis kits (Takara Bio USA, Cat#ab117152) according to the manufacturer's instructions. cDNA products were analyzed using qPCR with SYBR Select Master Mix (Life Technologies) on a Fluorescent quantitative PCR system (SLAN-96S, Shanghai Hongshi Medical Technology). The amplification process included an initial heating step at 95 °C for 2 min, followed by 40 cycles of 95 °C for 10 s, and 60 °C for 30 s, concluding with melt curve analysis to assess quality. The primer sequences for the human genes examined in this study are as follows: USP14 forward: TGTGCCTGAACTCAAAGATGC; USP14 reverse: ATATACTGCGCTGAAGCCATTT; ALKBH5 forward: CGGCGAAGGCTACACTTACG; ALKBH5 reverse: CCACCAGCTTTTGGATCACCA; FANCI forward: CCACCTTTGGTCTATCAGCTTC; FANCI reverse: CAACATCCAATAGCTCGTCACC; FANCD2 forward: TCAGACCCTGAGGAGACACC; FANCD2 reverse: ATGTCAATCCCCAGAAGCAG; RAD51 forward: GGAACTGCAACTCATCTGGG; RAD51 reverse: CATTGCCATTACTCGGTCCG; MKI67 forward: CTGCTTGTTTGGAAGGGGTA; MKI67 reverse: AGCCGTACAGGCTCATCAAT; GAPDH forward: CTGGGCTACACTGAGCACC; GAPDH reverse: AAGTGGTCGTTGAGGGCAATG.

### Immunohistochemical (IHC) analysis

All IHC analyses on the clinical specimens in this study were conducted under protocols approved by the medical ethics committee of Xiamen University. A total of 64 de-identified paraffin-embedded human glioma specimens were collected at Union Hospital of Tongji Medical College, Huazhong University of Science and Technology, China. These clinical glioma specimens were examined and diagnosed by pathologists at Union Hospital of Tongji Medical College, Huazhong University of Science and Technology. Further clinical information is detailed in Supplemental Table 1. The expression levels of MST4, p-ALKBH5, and USP14 proteins in different glioma tissues were analyzed using IHC. The experimental procedure followed the EnVisionTM + Dual Link System - HRP kit (Dako) protocol. DAB chromogenic sealing was performed after antibody combination experiments, and images were collected using an Olympus IXplore microscope for subsequent quantitative analysis conducted with ImagePlus. Percentage of positively stained cells was quantified and statistically analyzed as previously described following: 0 if 0% of the tumor cells exhibited positive staining, 1 for 0-1% of cells stained, 2 for 2-10% of cells stained, 3 for 11-30% of cells stained, 4 for 31-70% of cells stained and 5 for 71-100% of cells stained. In addition, the staining intensity was rated on a scale of 0-3: 0, no staining; 1, weak; 2, moderate; and 3, strong. The proportion and intensity scores were then combined to obtain a total score (range, 0-8).

### Seahorse assay

The extracellular acidification rate (ECAR) and oxygen consumption rate (OCR) were quantified using an XF96 extracellular flux analyzer (Agilent Technologies, Seahorse Bioscience, USA). For the OCR assessment, the cell culture medium was substituted with pre-warmed assay medium (Seahorse Bioscience) enriched with 1 mM pyruvate, 10 mM glucose, and 2 mM glutamine, and incubated at 37 °C in a non-CO_2_ environment for 1 h. Subsequently, Oligomycin (2 μM), FCCP (2 μM), Rotenone (1 μM), and Antimycin A (1 μM) were administered into the designated ports of a hydrated sensor cartridge. In the case of the ECAR assessment, the cell medium was replaced with pre-warmed assay medium containing 2 mM glutamine, and the microplate was incubated at 37 °C in a non-CO_2_ incubator for 1 h prior to the measurements. Glucose (10 mM), Oligomycin (2 μM), and 2-DG (50 mM) were sequentially injected. OCR and ECAR were evaluated to ascertain the cellular energy phenotype, with three measurements taken after each injection. After data collection, raw data were exported via Excel and plotted using GraphPad Prism.

### Polysome profile

Polysome profiling was performed by LC-Bio Technology CO., Ltd (hangzhou, China), with GEO number GSE285535. Briefly, ALKBH5 KO or control GSC 83 cells were subjected to cycloheximide (CHX) treatment at a concentration of 100 μg/mL for 15 min at 37 °C. Following treatment, cells were collected and resuspended in pre-cooled PBS supplemented with 100 μg/mL CHX. The cells underwent three washing cycles and were centrifuged to eliminate the supernatant. Subsequently, the cells were lysed and layered onto a sucrose gradient tube ranging from 10% to 45%, then centrifuged at 36,000 rpm using a Beckman SW-41Ti rotor for 3 h at 4 °C. Peak fractions were isolated based on A260 optical density using a gradient fractionation station. mRNAs associated with four or more ribosomes were extracted as polysome fractions and purified using TRIzol Reagent. Poly-A selected mRNAs were subsequently isolated and utilized for library preparation. RNA libraries were prepared by LC-Bio Technology CO., Ltd (hangzhou, China), followed by sequencing on the Illumina NovaSeq 6000 platform.

### Statistical analysis

Statistical analyses were conducted utilizing Microsoft Excel 2018 and GraphPad Prism (version 8.3.0) software. Experimental data are presented as the mean ± SEM of at least three biologically independent replicates or experiments per data point. To compare two groups, two-tailed Student's t-tests were employed unless indicated otherwise. To evaluate differences among more than two groups, we used the one-way ANOVA with Tukey's multiple comparisons test or two-way ANOVA test. Survival data were evaluated using the log-rank (Mantel-Cox) test and are represented as Kaplan-Meier survival curves.

## Supplementary Material

Supplementary figures and table.

## Abbreviations

ALKBH5: α-ketoglutarate-dependent dioxygenase alkb homologue 5
CHX: cycloheximide
CL: classical
CSCs: cancer stem cells
DGC: differentiated glioblastoma stem cells
DUBs: deubiquitylating enzymes
ECAR: extracellular acidification rate
GBM: glioblastoma
GSCs: glioblastoma stem cells
HR: homologous recombination
IR: ionizing radiation
IF: immunofluorescence
KD: knockdown
KO: knockout
MES: mesenchymal
MST4: mammalian sterile-20-like kinase 4
LC-MS: liquid chromatography-mass spectrometry
LGG: low-grade gliomas
NHA: normal astrocytes
NHEJ: non-homologous end joining
NPCs: normal neural progenitor cells
OCR: oxygen consumption rate
PN: proneural
PVDF: polyvinylidene fluoride
qPCR: quantitative real-time polymerase chain reaction
USP14: ubiquitin-specific protease-14
USPs: ubiquitin-specific proteases
