# Supplementary material for: USP14 modulates stem-like properties, tumorigenicity, and radiotherapy resistance in glioblastoma stem cells through stabilization of MST4-phosphorylated ALKBH5
Source: Theranostics. 2025 Jan 13;15(6):2293–314. doi: 10.7150/thno.103629 (PMC11840735; doi:10.7150/thno.103629)
Supplement: Supplementary file 1 — Supplementary figures and table. [file thnov15p2293s1.pdf]

Supplemental Information

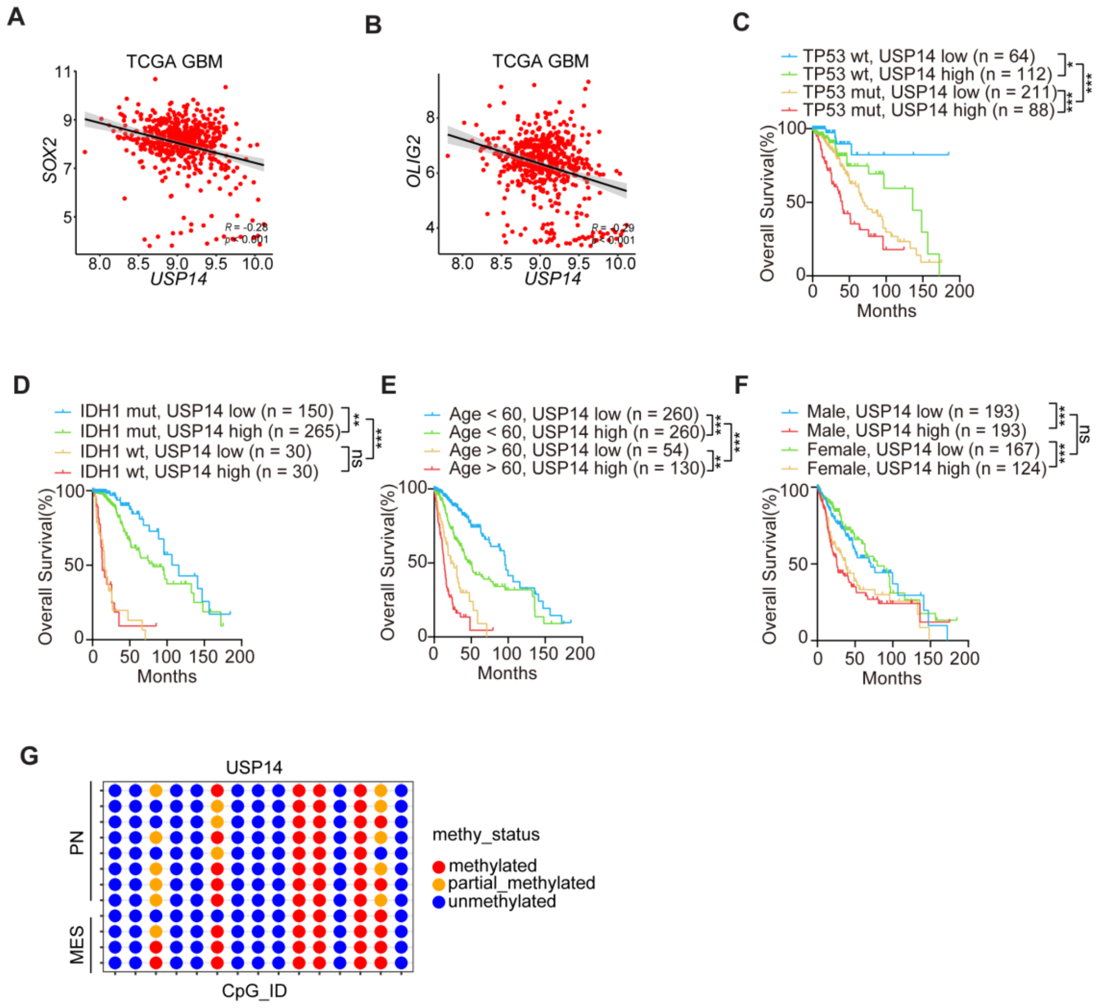

Supplementary Figure 1: Analyses of USP14 expression in the glioma.

(**A** and **B**) Pearson correlation between *USP14* and *SOX2* (A) and *OLIG2* (B) mRNA expression in the TCGA GBM datasets.

(**C-F**) Comprehensive multivariate analyses utilizing Kaplan-Meier survival curves were conducted to assess the relationship between USP14 expression and various factors, including TP53 status (C), IDH1 status (D), age (E, categorized as < or > 60 years), and sex (F), using the TCGA RNA-1 dataset encompassing both LGG and GBM.

(**G**) Direct bisulfite sequencing analysis of the USP14 promoter region was performed to assess the methylation status of individual CpG sites in PN or MES glioma stem cells (GSCs). The methylation index (MI) was calculated as the ratio of methylated CpG sites to the total number of CpG sites. Red circles indicate methylated CpG sites, yellow circles represent partial methylation, and blue

circles denote unmethylated CpG sites. The dataset is available under GEO accession number GSE90498.

Statistical analyses were determined by Pearson Correlation test (A, B), or log-rank test (C-F). $*p < 0.05$; $**p < 0.01$; $***p < 0.001$; ns, no significance.

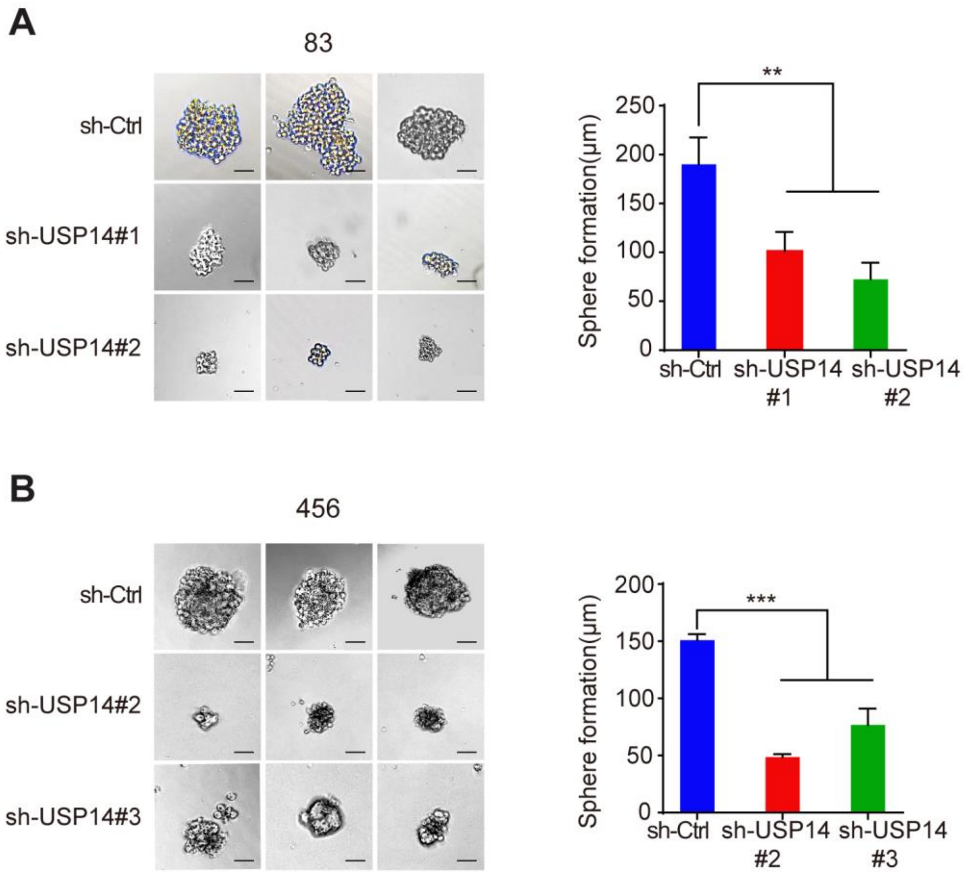

Supplementary Figure 2: USP14 is essential for the self-renewal capacity of GSCs.

(**A** and **B**) Left panel: Representative images of glioma spheroids derived from GSC 83 and 456 cell lines transduced with control shRNA (sh-Ctrl) and different shRNAs targeting USP14. Right panel: Quantitative analysis of the impact of USP14 knockdown on the sizes of GSC spheroids. Scale bars: 100 µm. Statistical significance was determined by one-way ANOVA with Tukey's multiple comparisons test (A and B). ** $p < 0.01$; *** $p < 0.001$.

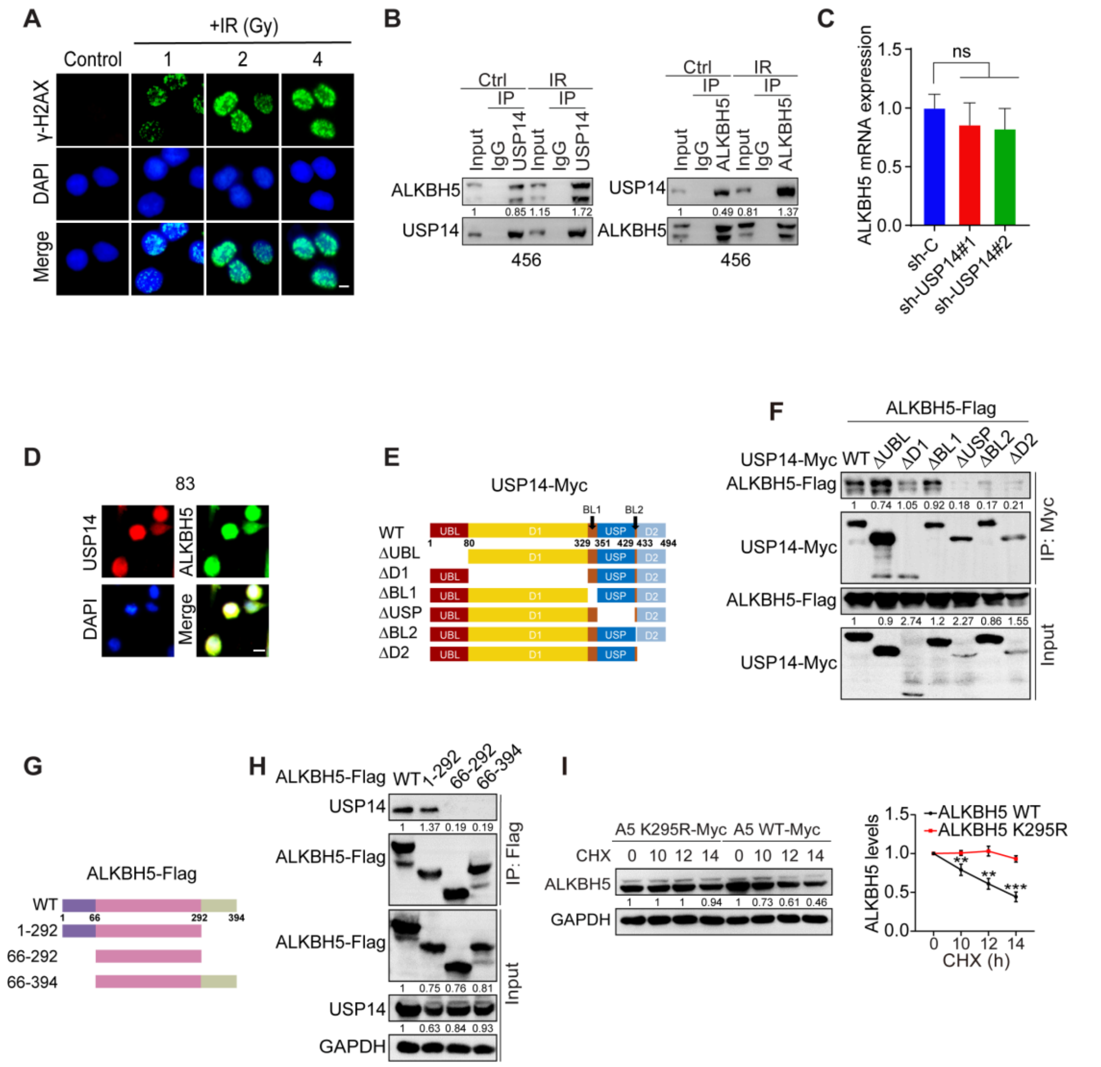

Supplementary Figure 3: USP14 mediates the stabilization of ALKBH5 via direct molecular interactions.

(**A**) Immunofluorescent staining (IF) for γH2AX foci formation in GSC 83 with or without indicated dose of irradiation (IR) treatment. Green, γH2AX; blue, DAPI. Scale bar, 5 μm.

(**B**) Immunoprecipitation followed by immunoblotting (IP-IB) analysis of USP14-ALKBH5 interaction in GSC 456 cells with or without IR treatment (2 Gy). IgG: immunoglobulin G.

(**C**) Quantitative PCR analysis of *ALKBH5* mRNA expression in GSC 83 cells with and without USP14 knockdown.

(**D**) IF of USP14 and ALKBH5 in GSC 83 cells. Scale bars, 10 μm.

(**E**) Schematic representation of USP14 truncation mutants used in this study.

(**F**) IP-IB analysis of the interaction between ALKBH5 and the indicated domains of USP14.

(**G**) Schematic representation of ALKBH5 truncation mutants used in this study.

(**H**) Co-IP analysis of the interaction between USP14 and the indicated ALKBH5 domains.

(**I**) IB analysis of ALKBH5 WT and K295R protein levels in HEK293T cells treated with 50 μg/mL cycloheximide (CHX) for indicated time periods. The relative intensities of ALKBH5 protein bands were quantified against untreated controls.

Statistical significance was determined by one-way ANOVA with Tukey's multiple comparisons test (C), or two-tailed Student's t-test (I). Data are representative of two to three independent experiments with similar results. All data presented in the bar graphs are expressed as means ± SEM. $^{**}p < 0.01$. $^{***}p < 0.001$. ns, no significance.

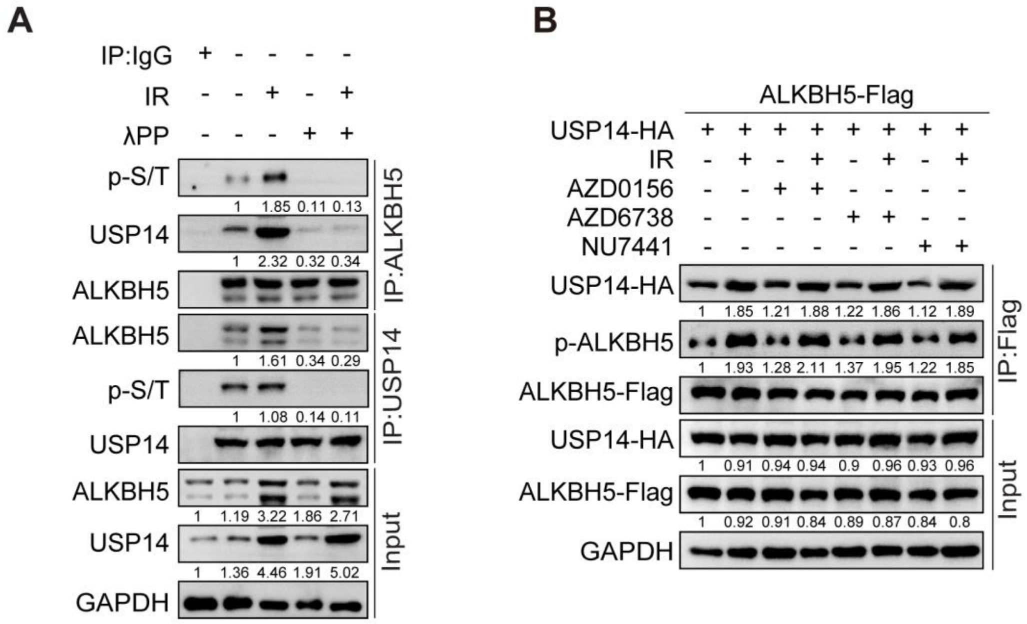

Supplementary Figure 4: Radiation induced phosphorylation of ALKBH5.

(**A**) IP-IB analysis of the interaction between ALKBH5 and USP14 in irradiation (IR)-treated GSC23 cells. GSC23 cells were harvested 48 hours after 4 Gy of IR treatment. The immunoprecipitated proteins were subsequently treated with λ phosphatase and subjected to immunoblotting analysis using the indicated antibodies.

(**B**) IP-IB analysis of the interaction between ALKBH5 and USP14, as well as the phosphorylation status of ALKBH5 in GSC23 cells with treatment of irradiation (IR), ATM inhibitor AZD0156, ATR inhibitor AZD6738, or DNA-dependent protein kinase (DNA-PK) inhibitor NU7441.

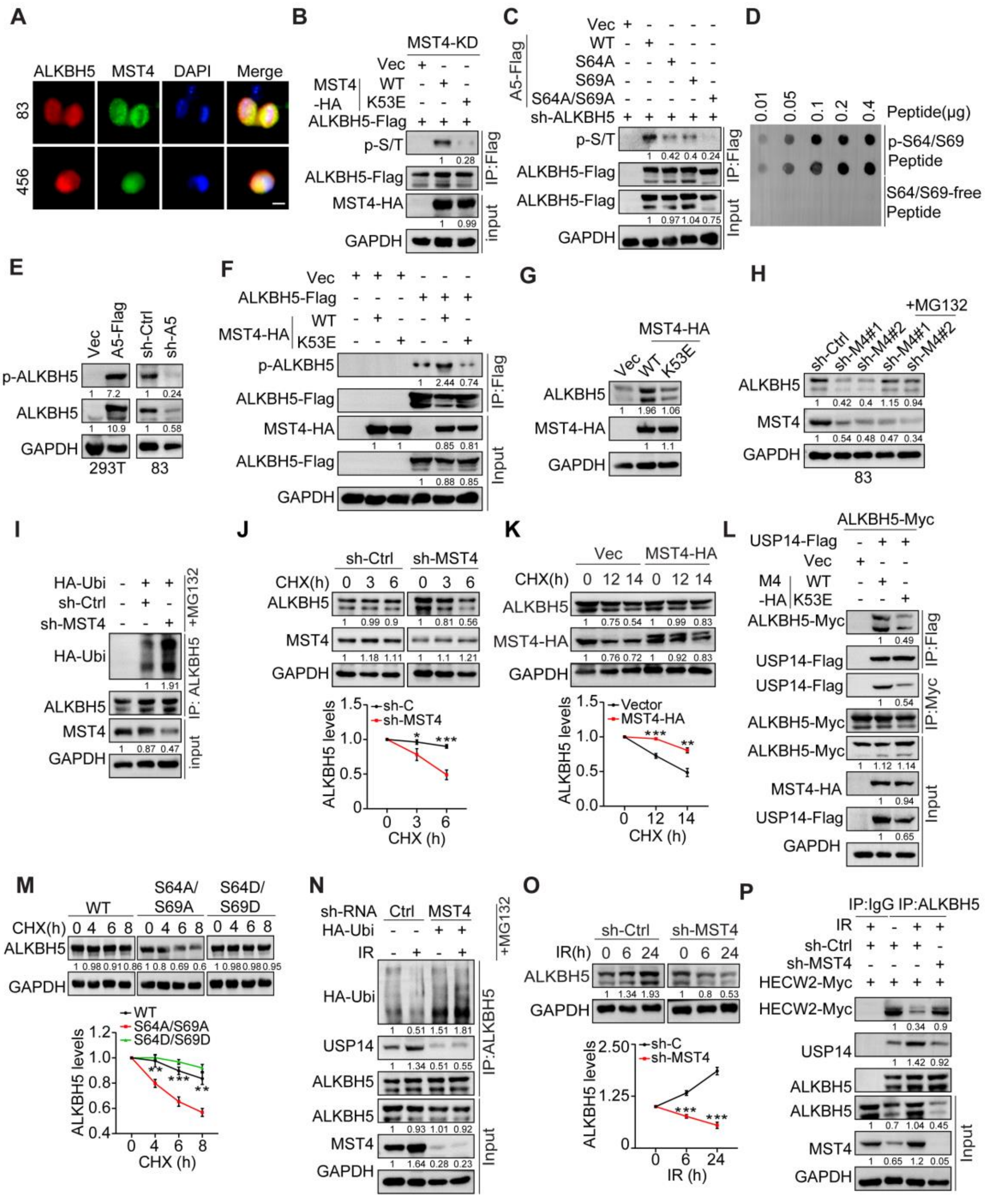

Supplementary Figure 5: MST4-mediated phosphorylation of ALKBH5 enhanced the stability of ALKBH5 proteins via USP14-dependent deubiquitination.

(**A**) IF analyses of GSC 83 and 456 cells using anti-MST4 and anti-ALKBH5 antibodies. Scale bars, 10 μm.

(**B**) IP-IB analysis of ALKBH5 phosphorylation in GSC 83 cells with MST4 knockdown (KD), re-expression with either wild-type (WT) MST4 or the kinase-inactive MST4 mutant K53E.

(**C**) IP-IB analysis of ALKBH5 phosphorylation in GSC 83 cells lacking ALKBH5, reconstituted with ALKBH5-WT, S64A, S69A, or S64A/S69A variants.

(**D**) The specificity analysis of human ALKBH5 phosphorylation antibody (anti-p-ALKBH5) with S64/69 unmodified (S64/69-free) and phosphorylated (p- S64/69) peptides.

(**E**) The specificity evaluation of anti-p-ALKBH5 was conducted through IB analysis of HEK293T cells expressing exogenous ALKBH5-Flag and GSC 83 cells with ALKBH5 KD.

(**F**) IP-IB analysis of ALKBH5 phosphorylation in HEK293T cells transfected with either MST4-HA-WT or mutant K53E, with or without the co-expression of ALKBH5-Flag.

(**G**) IB analysis for ALKBH5 and MST4 in HEK293T expressing either MST4-HA-WT or mutant K53E.

(**H**) GSC83 cells with or without MST4 KD were treated with 20 μM MG132 or DMSO for 6 hours. Cell lysates were then subjected to IB analysis for indicated proteins.

(**I**) GSC83 cells were transduced with HA–Ubi along with either shRNA-MST4 or sh-Ctrl, subsequently exposed to MG132 treatment. The resulting cell lysates were subjected to IP-IB analysis of ALKBH5 ubiquitination.

(**J** and **K**) IB analysis for ALKBH5 and MST4 in 293T cells with either MST4 knockdown (J) or overexpression (K). The cells were treated with 50 μg/mL cycloheximide (CHX) for the indicated time (upper). The levels of ALKBH5 protein were quantified (bottom).

(**L**) IP-IB analysis of ALKBH5-USP14 interactions in HEK293T cells transfected with either MST4-HA-WT or mutant K53E.

(**M**) IB analysis for ALKBH5 in HEK293T cells with ALKBH5 knockout, restored with ALKBH5-WT, S64A/S69A or S64D/S69D mutants. These cells were treated with 50 μg/mL CHX for indicated time (upper panel). The expression levels of ALKBH5 were quantified (lower panel).

(**N**) IP-IB analysis for ALKBH5 ubiquitination in GSC456 cells with indicated modifications and treatments.

(**O**) IB analysis for ALKBH5 in 293T cells with or without MST4 knockdown and 4 Gy of IR treatment (upper). The protein levels of ALKBH5 were quantified (bottom).

(**P**) IP-IB for indicated proteins in 293T cells with indicated modifications.

Statistical significance was determined by two-tailed Student's t-test (J, K and O), or one-way ANOVA with Tukey's multiple comparisons test (M). Data in bar or line graphs are mean ± SEM. $*p < 0.05$; $**p < 0.01$; $***p < 0.001$. Data are representative of two or three independent experiments with similar results.

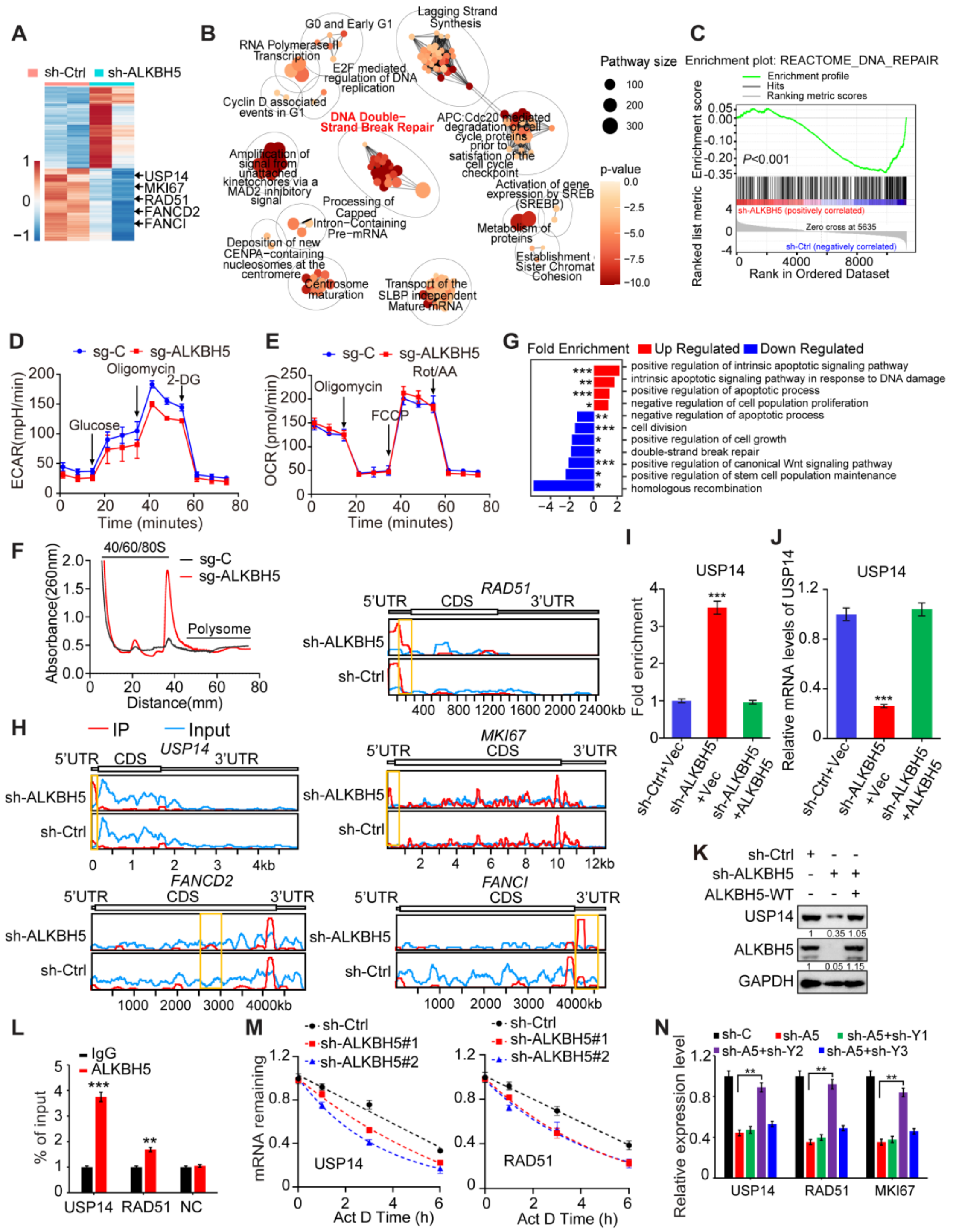

Supplementary Figure 6: ALKBH5 regulates the stability of USP14 and RAD51 in an m^{6}A-dependent manner.

(**A**) Heatmaps showing the top and bottom 50 most differentially expressed genes in GSCs expressing sh-Ctrl or sh-ALKBH5 (GEO: GSE87515).

(**B**) Enrichment map visualization of downregulated gene sets in GSCs after ALKBH5 knockdown.

(**C**) GSEA analysis demonstrating the upregulation of DNA double-strand break repair pathways in ALKBH5-depleted GSCs.

(**D**) ECAR of sg-Ctrl and sg-ALKBH5 GSC 83 cells.

(**E**) OCR of sg-Ctrl and sg-ALKBH5 GSC 83 cells.

(**F**) Polysome profiling of sg-Ctrl and sg-ALKBH5 GSC 83 cells.

(**G**) Gene Ontology analysis of transcripts exhibiting differential translation efficiency.

(**H**) IGV tracks displaying m^6A abundances of *USP14, FANCI, MKI67, FANCD2,* and *RAD51* transcript in ALKBH5-knockdowned and control cells.

(**I**) m^6A-RIP-qPCR analysis of m^6A enrichment on *USP14* mRNA in GSC 83 cells with indicated modifications.

(**J**) qRT-PCR assessment demonstrating the expression levels of *USP14* mRNA in GSC 83 cells with indicated modifications.

(**K**) IB analysis for ALKBH5 and USP14 in GSC 83 cells with indicated modifications.

(**L**) RIP-qPCR analysis of ALKBH5 binding to *USP14* and *RAD51* mRNA in GSCs. Primers of *USP14* or *RAD51* locates 5´ UTR around m^6A sites and primer NC in the distal region without m^6A site.

(**M**) Relative levels of *USP14* or *RAD51* in indicated GSCs were measured by qPCR, at the indicated time after 5 µg/mL actinomycin D treatment.

(**N**) qRT-PCR analysis of *USP14* and *RAD51* expression levels in GSCs with indicated modifications. A5: ALKBH5; Y1-3: YTHDF1-3.

Statistical significance was determined by two-tailed Student's t-test (H, K, M). Data in bar plots, and line graphs are means ± SEM. $*p < 0.05$; $**p < 0.01$; $***p < 0.001$; Data are representative of two to three independent experiments with similar results.

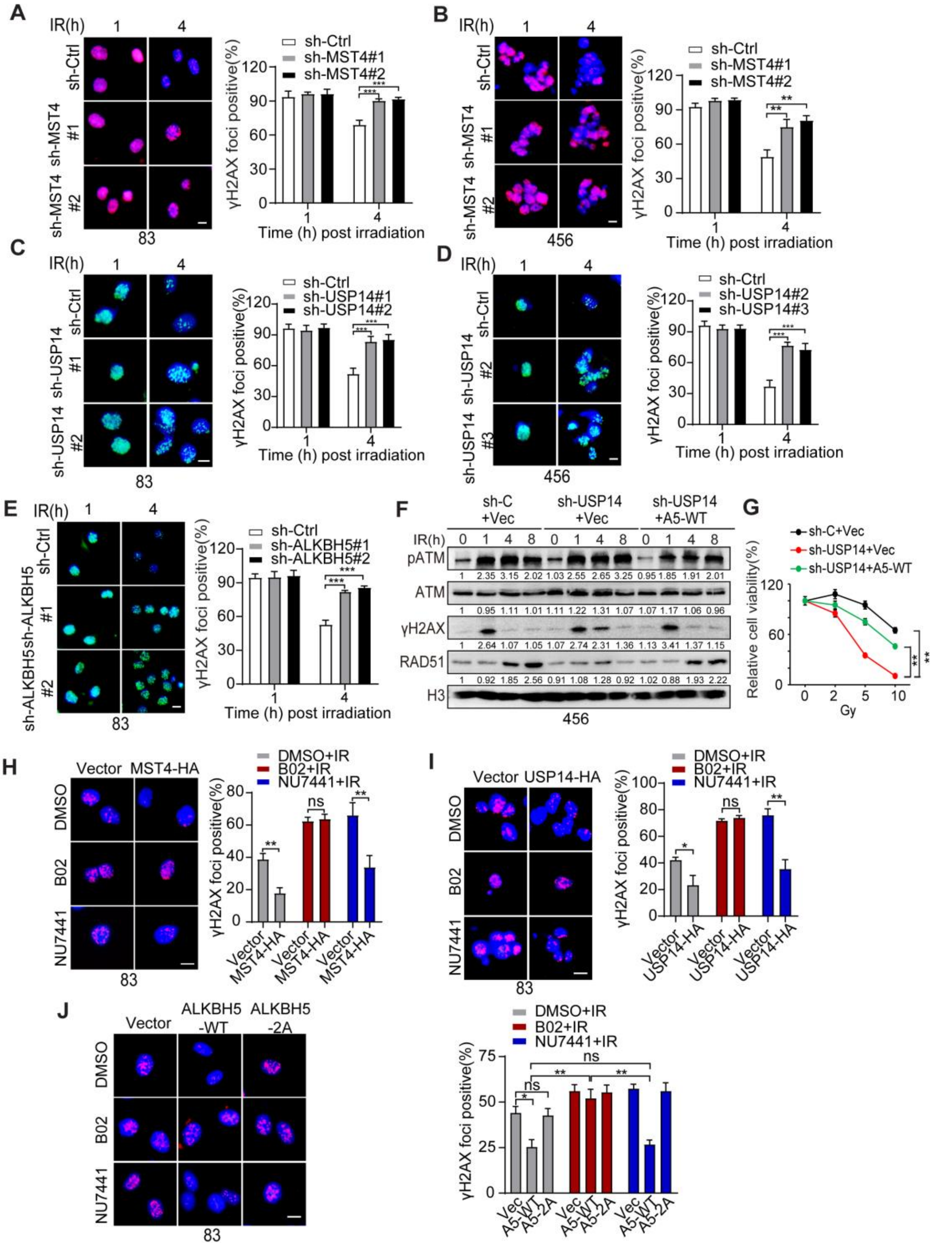

Supplementary Figure 7: USP14-MST4-ALKBH5 signalling axis regulates DNA damage response.

(**A**) to (**E**), Representative micrographs (left) and quantification data (right) for γH2AX foci formation in the indicated cells at 1 h and 4 h after IR treatment (4 Gy).

(**F**) IB analysis for indicated protein in GSC456 cells with indicated modifications and treatments.

(**G**) Cell viability for GSC456 cells with indicated modifications at day 5 after IR treatments.

(**H** and **I**) Representative micrographs (left) and quantification data (right) for γH2AX foci formation in GSC83 cells with or without overexpression of MST4 (H) or USP14 (I). Cells were treated with 4 Gy of IR, as well as B02, NU7441, or DMSO.

(**J**) Representative micrographs (left) and quantification data (right) for γH2AX foci formation in GSC 83 cells overexpressing the control vector, ALKBH5-WT or the S64A/S69A mutant (2A). The cells were treated with B02, NU7441, or DMSO, followed by exposure to IR (4 Gy).

Statistical significance was determined by one-way ANOVA with Tukey's multiple comparisons test (A-E and J), two-way ANOVA test (G), and two-tailed Student's t-test (H-I). Data in bar plots, and line graphs are means ± SEM. *$p < 0.05$; **$p < 0.01$; ***$p < 0.001$; ns, no significance. Data are representative of two to three independent experiments with similar results. Scale bars, 10 µm.

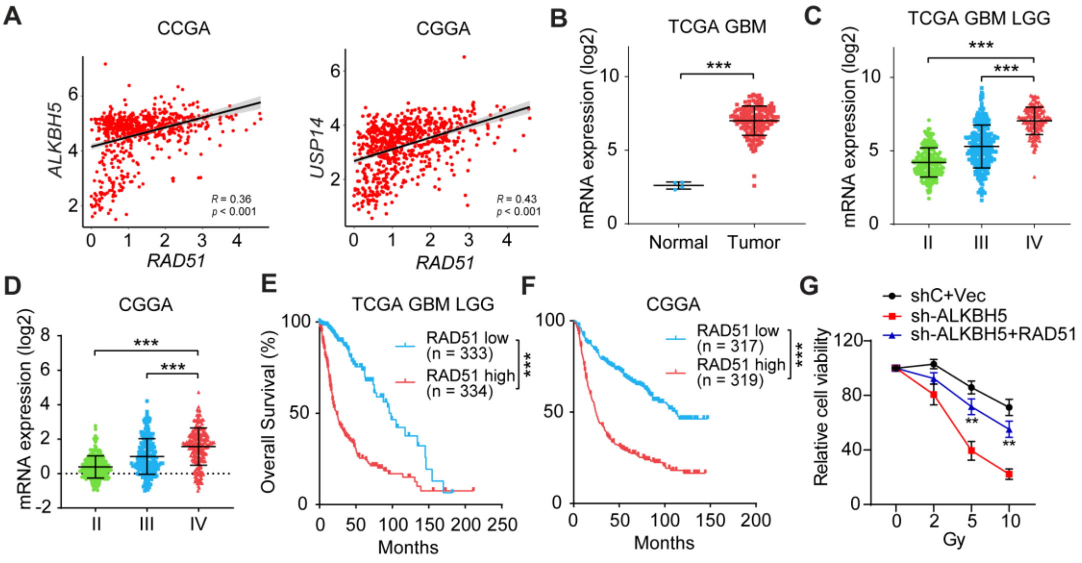

Supplementary Figure 8: RAD51 acts as a downstream effector of the USP14-MST4-ALKBH5 signalling pathway in the radioresistance of GSCs.

(**A**) The Pearson correlation analysis assessing the expression levels of ALKBH5 (left), USP14 (right) in relation to RAD51 within the CGGA LGG+GBM dataset. Both axes are scaled using log2 (TPM).

(**B**) Relative mRNA expression levels of *RAD51* in normal brain tissues and GBM samples were determined in TCGA dataset (***$p < 0.001$).

(**C** and **D**) Analyses of the TCGA (C) and CGGA (D) datasets for expressions of RAD51 between different grades of gliomas.

(**E** and **F**) Kaplan-Meier analyses of the TCGA (E) and CGGA (F) LGG+GBM dataset for RAD51 expression.

(**G**) Cell viability for the indicated GSC456 cells at day 5 after IR (5 Gy) treatments.

Data in bar or line graphs are mean ± SEM. **$p < 0.01$, and ***$p < 0.001$.

Statistical analyses were determined by Pearson Correlation test (A), two-tailed Student's t-tests (B), one-way ANOVA with Tukey's multiple comparisons test (C, D and G), log-rank test (E, F).

Data are representative of two or three independent experiments with similar results.

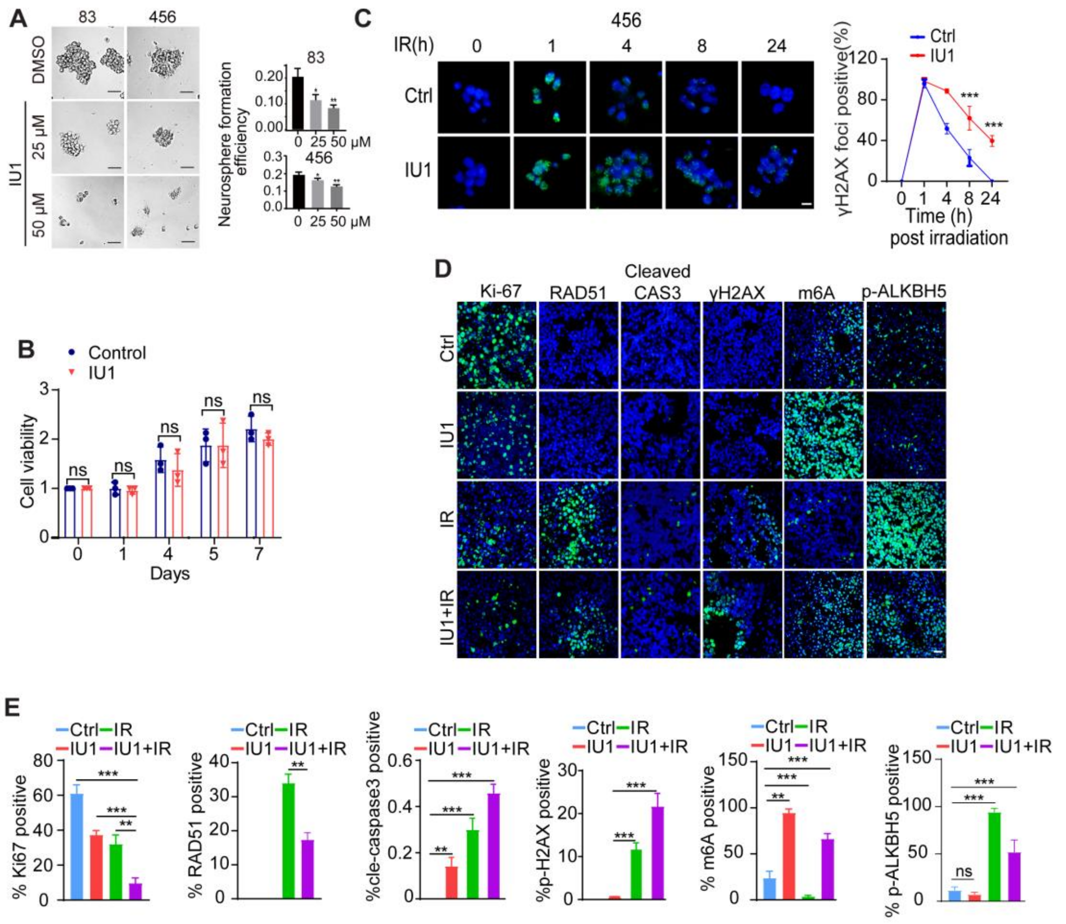

Supplementary Figure 9: The USP14 inhibitor IU1 attenuates GSC self-renewal and enhances the antitumoral effect of IR on GSCs.

(**A**) Neurosphere formation was assessed in GSC 83 and 456 cells treated with 25 μM or 50 μM IU1 for 7 days. Representative images are shown (left panel). Scale bars, 100 μm. Neurosphere formation efficiency (spheres/cells plated) was quantified (right panel).

(**B**) Cell viability for neural stem cells treated with 25 μM IU1 for indicated times.

(**C**) GSC 456 cells were subjected to treatment with or without 25 μM IU1, followed by exposure to 5 Gy of IR. After this, the cells were stained at indicated time points using antibodies against γH2AX. Cells exhibiting more than five γH2AX foci were classified as positive. Scale bars, 10 μm.

(**D**) IF staining for Ki-67, RAD51, cleaved caspase 3, γH2AX, m^{6}A, and p-ALKBH5 expression in brain sections with GSC 83 brain tumor xenografts with indicated treatments. Scale bars, 50 μm.

(**E**) Quantification of IF data in (D).

Statistical significance was determined by one-way ANOVA with Tukey's multiple comparisons test (A, E), or two-tailed Student's t-test (B, C). Data presented in bar or line graphs are expressed as mean ±SEM. *$p < 0.05$; **$p < 0.01$; ***$p < 0.001$; ns, no significance.

Data are representative of two or three independent experiments with similar results.

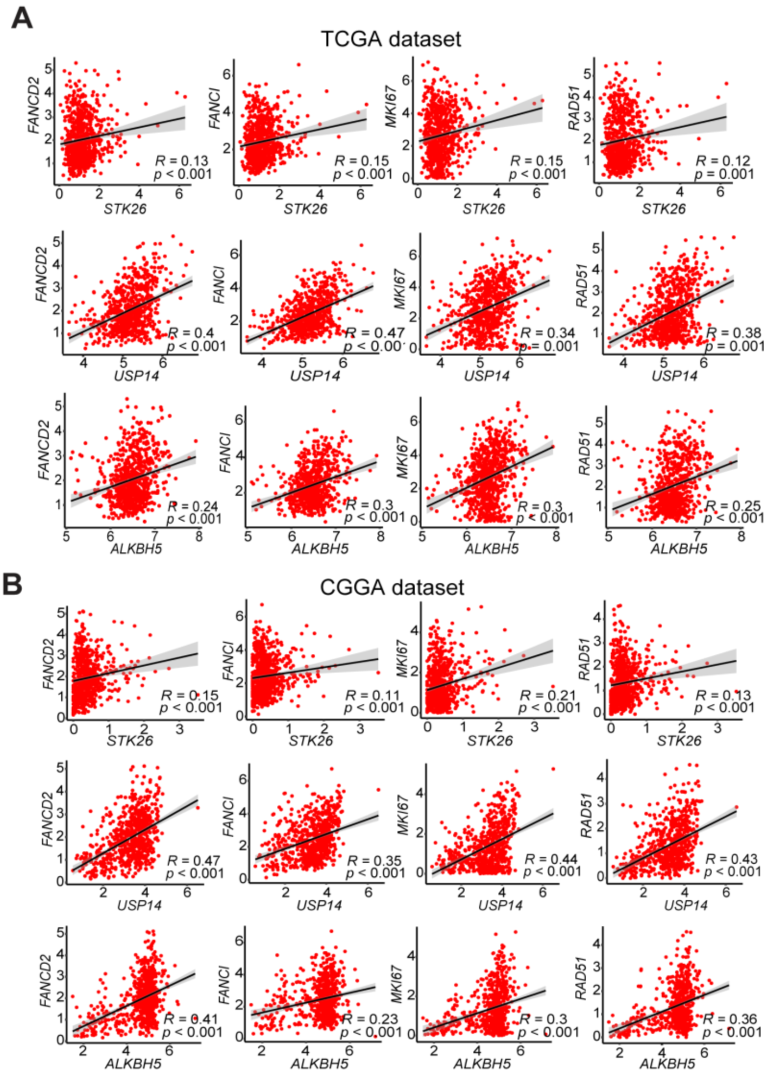

Supplementary Figure 10: ALKBH5 regulates DNA repair pathways.

(**A** and **B**) Pearson correlation analysis between MST4/STK26, USP14, or ALKBH5 and FANCI, FANCD2, MKI67, or RAD51 in the glioma datasets from TCGA (A) and CGGA (B). Statistical analyses were determined by Pearson Correlation test (A, B).

Supplementary Table 1: Patient clinical information.

No.	Age(yr)	Sex	Location	Pathological diagnosis	K_i-67
1	50	F	L T	GBM	25%
2	38	M	L T	GBM	60%
3	60	F	N/A	Gr III	15%
4	52	M	L F	GBM	15%
5	55	F	N/A	N/A	30%
6	29	M	R F	Gr III	30%
7	44	M	L T	Gr III	20%
8	19	F	L P	Gr II	<3%
9	N/A	F	N/A	N/A	N/A
10	33	F	N/A	N/A	3%
11	N/A	M	N/A	N/A	N/A
12	38	F	L F	Gr III	10%
13	39	M	R F	GBM	40%
14	60	M	R T	Gr I	<1%
15	59	F	N/A	N/A	N/A
16	21	F	R P	Gr III	30%
17	36	F	L F	Gr I	2%
18	34	F	L F	Gr III	15%
19	67	F	R F	N/A	N/A
20	61	M	R F	N/A	80%
21	51	F	L F	Gr II	3%
22	44	M	L F	Gr II	5%
23	67	F	R F	N/A	70%
24	33	M	L T	Gr III	20%
25	51	M	R F	GBM	30%
26	42	M	L T	GBM	15%
27	39	F	R T	Gr III	20%
28	62	F	N/A	N/A	N/A
29	52	M	L T	GBM	40%
30	34	F	R T	Gr II	5%
31	61	M	R T	GBM	25%
32	45	M	R T	GBM	20%
33	44	M	L F	Gr II	<3%
34	54	M	R T	GBM	20%
35	58	M	L F	Gr III	10%
36	34	M	R F	Gr II	<2%
37	60	M	R F	Gr II	2%
38	57	M	L T	Gr II	10%
39	73	M	R F	GBM	60%
40	34	F	R F	Gr II	<3%
41	53	F	L T	GBM	50%
42	33	M	L F	GBM	40%
43	53	M	L F	GBM	40%
44	74	M	L T	GBM	70%
45	29	M	R F	GBM	70%
46	68	M	N/A	N/A	N/A
47	50	F	L P	Gr III	20%
48	70	M	R T	GBM	20%
49	66	M	R T	Gr II	N/A
50	56	F	N/A	GBM	N/A
51	38	M	N/A	Gr II	N/A
52	52	M	R O	N/A	N/A

53	56	M	N/A	GBM	N/A
54	45	F	N/A	Gr II	N/A
55	56	M	N/A	Gr II	N/A
56	61	M	L F	N/A	N/A
57	71	F	R O	N/A	N/A
58	56	F	R P	N/A	N/A
59	56	M	N/A	N/A	N/A
60	54	F	N/A	Gr II	N/A
61	45	M	L P	N/A	N/A
62	39	M	N/A	Gr III	N/A
63	58	M	R P	N/A	N/A
64	66	M	L T	N/A	N/A

L F, left frontal lobe; L T, left temporal lobe; NA, not available; R F, right frontal lobe; R O, right occipital lobe; R T, right temporal lobe; L P, left parietal lobe; R P, right parietal lobe.
